# ERK Inhibition Promotes Engraftment of Allografts by Reprogramming T‐Cell Metabolism

**DOI:** 10.1002/advs.202206768

**Published:** 2023-04-04

**Authors:** Xiaosheng Tan, Changxing Qi, Xiangli Zhao, Lingjuan Sun, Mi Wu, Weiguang Sun, Lianghu Gu, Fengqing Wang, Hao Feng, Xia Huang, Bin Xie, Zhengyi Shi, Peiling Xie, Meng Wu, Yonghui Zhang, Gang Chen

**Affiliations:** ^1^ Institute of Organ Transplantation Tongji Hospital Tongji Medical College Huazhong University of Science and Technology Wuhan Hubei Province 430030 P. R. China; ^2^ Key Laboratory of Organ Transplantation Ministry of Education Chinese Academy of Medical Sciences Wuhan Hubei Province 430030 P. R. China; ^3^ NHC Key Laboratory of Organ Transplantation Chinese Academy of Medical Sciences Wuhan Hubei Province 430030 P. R. China; ^4^ Key Laboratory of Organ Transplantation Chinese Academy of Medical Sciences Wuhan Hubei Province 430030 P. R. China; ^5^ Hubei Key Laboratory of Natural Medicinal Chemistry and Resource Evaluation School of Pharmacy Tongji Medical College Huazhong University of Science and Technology Wuhan Hubei Province 430030 P. R. China; ^6^ Department of Immunology School of Basic Medicine Tongji Medical College Huazhong University of Science and Technology Wuhan Hubei Province 430030 P. R. China

**Keywords:** allograft rejection, extracellular regulated protein kinases (ERK), lycorine, metabolism, mitochondria

## Abstract

Extracellular regulated protein kinases (ERK) signaling is a master regulator of cell behavior, life, and fate. Although ERK pathway is shown to be involved in T‐cell activation, little is known about its role in the development of allograft rejection. Here, it is reported that ERK signaling pathway is activated in allograft‐infiltrating T cells. On the basis of surface plasmon resonance technology, lycorine is identified as an ERK‐specific inhibitor. ERK inhibition by lycorine significantly prolongs allograft survival in a stringent mouse cardiac allotransplant model. As compared to untreated mice, lycorine‐treated mice show a decrease in the number and activation of allograft‐infiltrated T cells. It is further confirmed that lycorine‐treated mouse and human T cells are less responsive to stimulation in vitro, as indicated by their low proliferative rates and decreased cytokine production. Mechanistic studies reveal that T cells treated with lycorine exhibit mitochondrial dysfunction, resulting in metabolic reprogramming upon stimulation. Transcriptome analysis of lycorine‐treated T cells reveals an enrichment in a series of downregulated terms related to immune response, the mitogen‐activated protein kinase cascade, and metabolic processes. These findings offer new insights into the development of immunosuppressive agents by targeting the ERK pathway involved in T‐cell activation and allograft rejection.

## Graphical abstract

Extracellular regulated protein kinases (ERK) pathway is highly activated in graft‐infiltrating T cells. Lycorine, an amaryllidaceae alkaloid extracted from the traditional Chinese medicine Zephyranthes candida, presents to be a novel ERK‐specific inhibitor and prolongs engraftment of allografts. Mechanistically, lycorine treatment induces mitochondrial dysfunction in T cells, which leads to T cell hyporesponsiveness to stimuli.

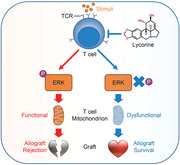

## 1 Introduction

Immunosuppression is one of the mainstay therapeutic strategies for treating autoimmune disorders, immune‐mediated inflammation, and allograft rejection.^[^ ^1^ ^]^ T‐cell activation plays a central pathogenic role in these situations through a variety of mechanisms, including the production of proinflammatory cytokines, expression of death receptor ligands, and cytotoxicity.^[^ ^2^ ^]^ The T cell receptor (TCR)‐peptide‐major histocompatibility complex (pMHC) interaction (signal 1) and costimulatory receptor engagement (signal 2) stimulate T cell activation through the calcium‐calcineurin pathway, the rat sarcoma (RAS)‐mitogen‐activated protein kinase (MAPK) pathway, and the nuclear factor pathway, leading to the expression of interleukin‐2 (IL‐2) and other cytokines.^[^ ^3^ ^]^ Thus, the development of immunosuppressive agents against T‐cell activation remains an important issue.

Given that MHC‐mismatched alloimmune responses represent one of the strongest T‐cell‐mediated responses, prevention of allograft rejection appears to require more potent immunosuppressive strategies. The most commonly used strategy is long‐term immunosuppressive maintenance therapy with drugs that inhibit T‐cell activation, proliferation, and proinflammatory function.^[^ ^4^ ^]^ In recent decades, many chemical compounds and antibodies have been selected for drug development on the basis of their ability to affect T‐cell activation pathways.^[^ ^5^ ^]^ Immunosuppressants routinely used in current clinical practice include calcineurin inhibitors (e.g., cyclosporin A and tacrolimus), inosine‐5’‐monophosphate dehydrogenase inhibitors (e.g., mycophenolate mofetil), mammalian target of rapamycin inhibitors (e.g., rapamycin), monoclonal antibody against IL‐2 receptor (e.g., basiliximab), and costimulatory receptor inhibitors (e.g., belatacept).^[^ ^6^ ^]^ In addition, protein kinase C inhibitors (e.g., sotrastaurin) and Janus kinase 3 inhibitors (e.g., CP‐690550) are undergoing or have been evaluated in clinical trials.^[^ ^7^ ^]^ Despite the tremendous successes achieved in organ transplantation as a result of the currently available immunosuppressive drugs, long‐term outcomes remain unsatisfactory.^[^ ^8^ ^]^ How to promote long‐term graft survival is still a challenging basic problem. Therefore, the study of new intervention targets of T cell activation and the development of corresponding immunosuppressive agents have broad application to the field of organ transplantation.

MAPKs, including the extracellular signal‐regulated kinases 1/2 (ERK1/2), c‐Jun N‐terminal kinase (JNK), and p38 MAP kinase, are a family of protein kinases whose function and regulation have been conserved throughout the evolution, from unicellular organisms (such as brewers’ yeast) to complex organisms (including humans).^[^ ^9^ ^]^ MAPKs can phosphorylate specific serines and threonines of target protein substrates and regulate cellular activities such as gene expression, mitosis, motility, metabolism, and programmed cell death.^[^ ^10^ ^]^ It has been documented that ERK1/2 signaling plays a crucial role in T‐cell activation, which is initially mediated by ZAP70, the tyrosine kinase of Syk family.^[^ ^11^ ^]^ Activation of ERK1/2 results in the transcription of c‐Fos and JunB, whose protein products heterodimerize to form the Activator protein‐1 (AP‐1) complex and bind to gene enhancers to stimulate the transcription of IL‐2 and many other genes.^[^ ^12^ ^]^ In addition, ERK1/2 signaling has been reported to be involved in T‐cell differentiation, metabolic regulation, and homeostasis.^[^ ^13^ ^]^ Previous studies have highlighted that finding that inhibition of MAP kinase 1/2 (MEK1/2), the kinase upstream of ERK1/2, leads to a reduction in phosphorylated ERK1/2, and T‐cell immunosuppression, and also has protective effects in inflammatory disease, graft‐versus‐host disease, and allograft rejection.^[^ ^14^ ^]^ However, little attention has been paid to the role of ERK signaling in allograft rejection, or to the development of drugs that specifically target ERK to inhibit immune activation and promote engraftment.

In the present study, we have used a stringent mouse cardiac allotransplant model to investigate the activation status of ERK signaling in recipient T cells. Using surface plasmon resonance (SPR) technology, we identified a specific inhibitor of ERK. We have also explored the effects of ERK suppression on T‐cell activation in vitro and in vivo. Furthermore, given the relationship between ERK and T‐cell metabolism, we also evaluated the changes in cellular energy metabolism in T cells in response to ERK inhibition.

## 2 Results

### 2.1 The ERK Pathway Is Activated in T Cells during Allograft Rejection

**Figure 1:**
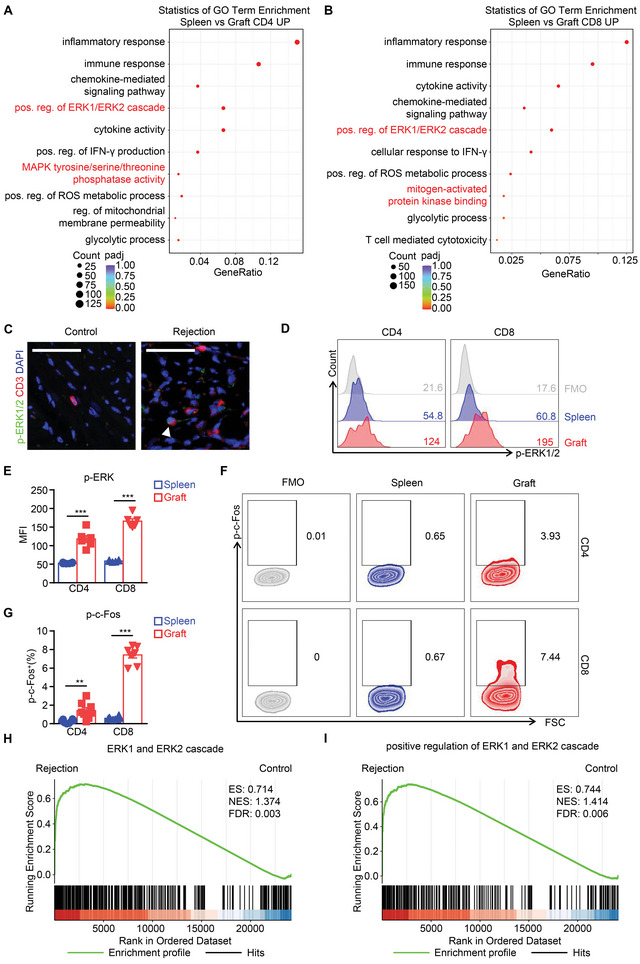
ERK signaling is activated in T cells during allograft rejection. A,B) Scatter plot showing the Gene Ontology (GO) enrichment results in the upregulated genes in graft‐infiltrating A) CD4^+^ T cells and B) CD8^+^ T cells. The dot size indicates the relative number of differentially expressed genes contained in the GO terms and the shade of the dots indicates the adjusted *P* value (padj) of the enrichment. C) Immunofluorescent staining of phosphorylated ERK1/2 (p‐ERK1/2, green) and CD3 (red) in graft sections from syngeneic (Control) or allogenic (Rejection) transplantation. D) Representative zebra plot and E) statistical graph for intranuclear phosphorylated ERK1/2 (p‐ERK1/2) levels in splenic and graft‐infiltrating T cell subsets. Fluorescent‐minus‐one (FMO) samples were used as isotype controls. F) Representative zebra plot and G) statistical graph for intranuclear phosphorylated c‐Fos (p‐c‐Fos) levels in splenic and graft‐infiltrating T cell subsets. H,I) Gene Set Enrichment Analysis (GSEA) shows an enrichment of ERK1/2‐related pathway up‐ and downregulated genes in the transcriptome of T cells from a rejected renal allograft. Data are shown as means ± SEM of at least three independent experiments. **P < 0.01, and ***P < 0.001, Student's *t‐*test.

Despite the need for sustained ERK activation during T cell activation, phosphorylation of ERK1/2 in response to TCR stimulation has been reported to be rapid and transient.^[^ ^15^ ^]^ To determine whether the ERK pathway is involved in alloreactive T cell activation, we compared the differences in downstream transcript changes between graft‐infiltrating and splenic T cells using a fully MHC‐mismatched (Balb/c to C57BL/6) murine heterotopic cardiac transplant model. By using Gene Ontology (GO) analysis of the upregulated genes in graft‐infiltrating T cell group, we enriched 160 terms in CD4^+^ T cells and 87 terms in CD8^+^ T cells with significant difference (padj < 0.05) (Table S1A,B, Supporting Information). Among which, a series of genes related to positive regulation of the ERK1/ERK2 cascade were upregulated in both graft‐infiltrating CD4^+^ and CD8^+^ T cells (**Figure** **1**A,B and Figure S1A, Supporting Information). Gene set enrichment analysis (GSEA) further revealed upregulation of ERK1/2 and MAPK‐related pathways in graft‐infiltrating T cells (Figure S1B,C, Supporting Information). By immunofluorescent staining, we observed colocalization of phosphorylated ERK1/2 and CD3 in rejected allograft sections but not in syngeneic grafts (Figure 1C). Flow cytometry detected increased mean fluorescence intensity of phosphorylated ERK1/2 in both graft‐infiltrating CD4^+^ and CD8^+^ T cells (Figure 1D,E). We further measured the phosphorylated c‐Fos levels in T‐cell subsets that act downstream of the activation of the ERK signaling pathway. The phosphorylation of c‐Fos was elevated in both CD4^+^ and CD8^+^ T cells isolated from the rejected allografts (Figure 1F,G). Consistent with our results, experimental data reported by others have shown that the “ERK1 and ERK2 cascade” pathway and the “positive regulation of ERK1 and ERK2 cascade” pathway were enriched in T cells isolated from rejected renal allografts by GSEA(Figure 1H,I).^[^ ^16^ ^]^ All the evidence suggests that the activation of ERK signaling is sustained in alloreactive T cells during allograft rejection.

### 2.2 Lycorine Is a Specific Inhibitor of ERK Activation in T Cells

**Figure 2:**
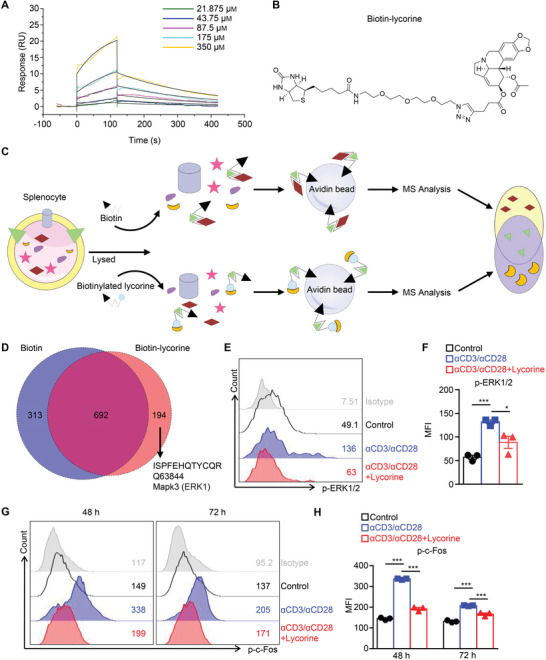
Identification lycorine as an ERK‐specific inhibitor. A) In SPR assays, recombinant human ERK1 protein was immobilized on a BIAcore CM5 sensor chip, followed by analysis its interaction with lycorine at the indicated concentrations. B) Structure of biotinylated lycorine. C) The schematic depicts the procedure for the affinity‐based approach to identify target(s) of lycorine from protein extracts derived from murine splenocytes. MS, mass spectrometry. D) The Venn diagram shows the percentages of overlapping and unique proteins pulled‐down by biotin and biotinylated lycorine. E,F) Flow cytometry staining for phosphorylated‐ERK1/2 (p‐ERK1/2) in splenocytes without stimulation, with anti‐CD3/anti‐CD28 bead stimulation, or with anti‐CD3/anti‐CD28 beads stimulation in the presence of lycorine (200 nm) for 15 min (*n* = 3 for each group). E) Representative histogram with mean fluorescence intensity and F) bar graph with mean ± SEM. G–H) Flow cytometry staining for phosphorylated‐c‐Fos (p‐c‐Fos) in CD4^+^ cells receiving the various treatments (*n* = 3 for each group), after 48 or 72 h later. G) A representative histogram with mean fluorescence intensity and H) bar graph are shown. Data are shown as means ± SEM and from one of three independent experiments. *P < 0.05, and ***P < 0.001, Student's *t‐*test.

To further investigate the role of ERK signaling in allograft rejection and T‐cell activation, we explored small‐molecule inhibitors of the ERK protein. We investigated natural products of traditional Chinese herbs, which can be useful compounds for drug development. Using SPR technology, we were able to identify lycorine, an amaryllidaceae alkaloid extracted from *Zephyranthes candida* and showed that this alkaloid binds the ERK1 protein with an estimated dissociation constant (KD) value of 156 µm and an association rate constant (ka) value of 24.28 m ^−1^ s^−1^ (**Figure** **2**A). To further validate this interaction, we synthesized biotinylated lycorine and incubated it with splenocyte lysates, pulled the resulting complexes down with streptavidin gel, and then analyzed them by mass spectrometry (MS) (Figure 2B,C). A total of 886 and 1005 peptides were identified that bound to biotinylated lycorine and biotin, respectively (Figure 2D). In the biotinylated lycorine group, only 194 peptides were specific, including ERK1 peptide (Mapk3) and 49 other peptides that could directly or indirectly interact with Mapk3 (Figure 2D and Figure S2, Supporting Information).

We then examined ERK phosphorylation after TCR‐dependent T‐cell activation in the absence and presence of lycorine. We found that phosphorylated ERK1/2 levels were markedly elevated in response to anti‐CD3/anti‐CD28 stimulation, but the levels were significantly decreased in splenocytes that had been pretreated with lycorine (200 nm; Figure 2E,F). Western blotting results showed that anti‐CD3/anti‐CD28‐induced T‐cell activation led to the phosphorylation of a series of protein kinases (e.g., ZAP70, PLC*γ*1, SLP‐76, p38, and ERK1/2). However, in the presence of lycorine, phosphorylation of ERK1/2 was reduced (Figure S3A, Supporting Information). In addition, lycorine treatment did not reduce the phosphorylation of MEK1/2 in CD4^+^ T cell with anti‐CD3/anti‐CD28 stimulation (Figure S3B,C, Supporting Information). A previous study has demonstrated that changes in the ERK1/2 conformational distribution in response to various inhibitor binding modes can affect molecular recognition by the phosphorylated molecule.^[^ ^17^ ^]^ With the help of virtual docking techniques, we found that lycorine can occupy the second pocket in ERK1 when it binds ASTX029, a phosphorylation and catalytic activity modulator of ERK1/2.^[^ ^18^ ^]^ Lycorine may alter the conformation of the target protein, in which Tyr36 becomes tucked under the loop and loses its *π*‐*π* stacking interaction with Tyr64. The additional hydroxy group in lycorine also afforded additional potency and bridged a charge‐charge interaction between Arg67 and Asp167 (Figure S3D, Supporting Information). Therefore, the downstream phosphorylation of c‐Fos was also reduced in the lycorine‐treated group when compared to the untreated group (Figure 2G,H). Together, these results indicate that lycorine can directly bind ERK1 and inhibit ERK phosphorylation, thereby inactivating the ERK pathway.

### 2.3 Lycorine Promotes Engraftment and Inhibits ERK Phosphorylation in a Stringent Mouse Cardiac Allotransplant Model

**Figure 3:**
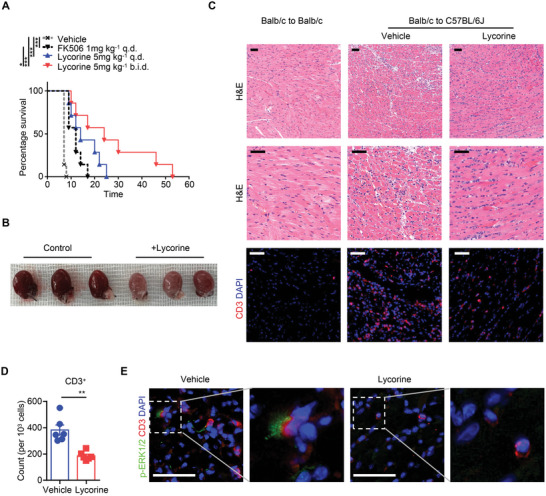
Inhibition of ERK activation by lycorine prolongs graft survival in heart transplanted mice. Recipient mice (C57BL/6) were intraperitoneally injected with lycorine for 3 d before heart transplantation. Hearts from Balb/c mice were transplanted to C57BL/6 mice. Allografts were harvested for analysis at the time of rejection or at the indicated time points. As a control, recipient mice received only an injection of the same volume of PBS (Vehicle). A) The kinetics of cardiac allograft survival rates for all study groups are shown (*n* = 7 for each group). B–D) Allografts were harvested at day 7 post‐transplant. B) Gross observation of allografts in the PBS‐treated and lycorine‐treated (5 mg kg^−1^, b.i.d.) groups. C) Hematoxylin and eosin (H&E) staining was performed to assess pathological changes (upper and middle panel). For measurement of the T‐cell infiltration in allografts from each group, immunofluorescence for CD3 was performed (lower panel). Scale bars, 50 µm. D) Quantitative plots for CD3 staining cell counts per 10^3^ cells in graft sections from each group (*n* = 6). E) Colocalization of phosphorylated ERK1/2 (p‐ERK1/2) and CD3 in graft sections from PBS‐ or lycorine‐treated recipients. Scale bars, 50 µm. Data are shown as means ± SEM and pooled from at least three independent experiments.*P < 0.05, and ***P < 0.001, A) Log‐rank (Mantel‐Cox) test and D) Student's *t‐*test.

To investigate whether lycorine is effective in suppressing allograft rejection and prolonging cardiac allograft survival, we injected lycorine intraperitoneally into C57BL/6 recipient mice daily, beginning 3 d before transplantation and continuing until the heart graft was found to have stopped beating on abdominal palpation. As a control, recipient mice received phosphate buffer saline (PBS) or tacrolimus (FK506). Cardiac allograft arrest occurred at 7–8 d after transplantation in PBS‐treated recipients. In contrast, treatment with either lycorine (5 mg kg^−1^, q.d. or b.i.d.) or FK506 (1 mg kg^−1^, q.d.) significantly prolonged allograft survival (**Figure** **3**A). The median graft survival time was 14 d in mice treated with low‐dose lycorine and 24 d in mice treated with high‐dose lycorine. More impressively, graft survival was significantly longer in the high‐dose lycorine treatment group than in the FK506 treatment group (24 vs 12 d, P < 0.05). Next, cardiac allografts from each group were harvested at 7 d after transplantation. Gross observation showed that the allografts in the PBS control group were dark red and enlarged in size, and the blood vessels on the surface were blurred. However, allografts in the high‐dose lycorine treated group had almost normal color, size, and vascular structure (Figure 3B). Hematoxylin and eosin (HE) staining and immunofluorescent staining for CD3 were performed to assess the pathological changes associated with acute allograft rejection. Allografts in the PBS control group developed severe acute rejection on day 7 after transplantation, mainly characterized by a diffuse mononuclear cell infiltration, myocardial interstitial edema, myocardial hemorrhage, and CD3^+^ T‐cell infiltration. In contrast, these pathological changes were significantly attenuated in the heart allografts from the high‐dose lycorine treated group 7 d after transplantation (Figure 3C,D). Unlike the PBS control group, colocalization of phosphorylated ERK1/2 and CD3 was absent from the tissue sections of the grafts from the high‐dose lycorine‐treated group (Figure 3E). These data demonstrate that lycorine can inhibit ERK phosphorylation and prolong allograft survival in vivo.

### 2.4 Lycorine Treatment Inhibits the Infiltration and Function of T Cells in Cardiac Allografts

**Figure 4:**
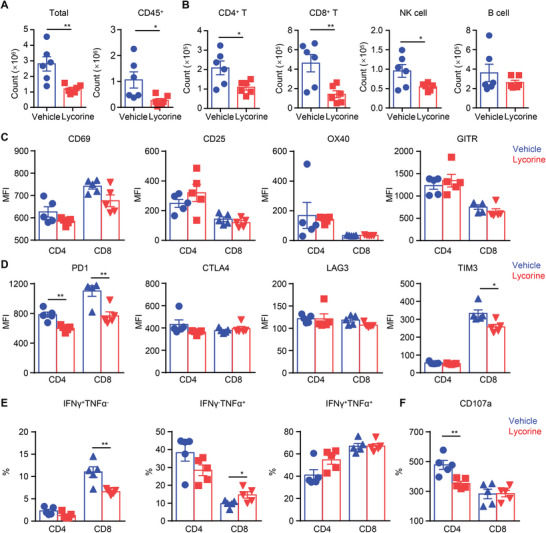
Lycorine treatment inhibits T cell infiltration and function in cardiac allografts. On day 7 posttransplant, allograft‐infiltrated leukocytes were isolated from PBS‐treated mice (Vehicle) or lycorine‐treated mice (5 mg kg^−1^, b.i.d. Lycorine) were isolated and analyzed by flow cytometry. A) Counts of allograft‐infiltrated cells and CD45^+^ cells in each group (*n* = 6). B) Numbers of allograft‐infiltrated CD4^+^ T cells, CD8^+^ T cells, B cells and NK cells (*n* = 6). C) The expression of membrane CD69, CD25, OX40, and GITR on the surface of allograft‐infiltrated CD4^+^ T cells and CD8^+^ T cells was measured (*n* = 5). Values for mean fluorescence intensity (MFI) are shown. D) Bar graphs depict MFI values for PD1, CTLA4, LAG3, and TIM3 on T‐cell subsets (*n* = 5). E) Allograft‐infiltrated leukocytes were stimulated with a Cell Stimulation Cocktail (plus protein transport inhibitors) and intracellular staining was performed. Percentages of IFN‐*γ* ^+^ and TNF‐*α* ^+^ T‐cell subsets are shown (*n* = 5). F) Levels of CD107a expression on allograft‐infiltrating CD4^+^ T cells and CD8^+^ T cells from each group (*n* = 5). The graphs show the means ± SEM of three independent experiments. *P < 0.05, and **P < 0.01, Student's *t‐*test.

To determine the inhibitory effect of ERK inhibition on alloreactive T cells, we isolated and purified leukocytes from allografts harvested 7 d after transplantation and measured the extent of the cell infiltrates, the levels of activation‐related markers, and cytokine‐producing capacity. Lycorine treatment (5 mg kg^−1^, b.i.d.) significantly reduced the amount of CD45^+^ leukocyte infiltrate, as well as of T‐cell subsets (CD4^+^, CD8^+^) and natural killer (NK) cells in the grafts when compared to the PBS control group (**Figure** **4**A,B). Even though the expression of activation‐related CD69, CD25, OX40, and glucocorticoid‐induced tumor necrosis factor (TNF) receptor (GITR) in the T cells did not differ significantly between the two groups, the lower PD1 and TIM3 levels we observed may still suggest that T‐cell activation was insufficient after lycorine treatment (Figure 4C,D). We further used a cocktail of phorbol 12‐myristate 13‐acetate (PMA), ionomycin (ION), and brefeldin A (BFA) to measure the cytokine‐producing capacity of graft‐infiltrating T cells. We found that the percentage of interferon (IFN)‐*γ* ^+^TNF‐*α* ^−^ CD8^+^ T cells was significantly lower in the lycorine‐treated group than in with the PBS control group (Figure 4E). However, the proportion of TNF‐*α*‐producing T cells was comparable between the two groups (Figure 4E). Next, we assessed degranulation by measuring CD107a expression in the CD4^+^ T cells and CD8^+^ T cells. A reduction in surface CD107a expression was detected in the graft‐infiltrating CD4^+^ T cells, but not in CD8^+^ T cells (Figure 4F).

### 2.5 Lycorine Suppresses Anti‐CD3/Anti‐CD28‐Induced T‐Cell Proliferation and Cytokine Production In Vitro

**Figure 5:**
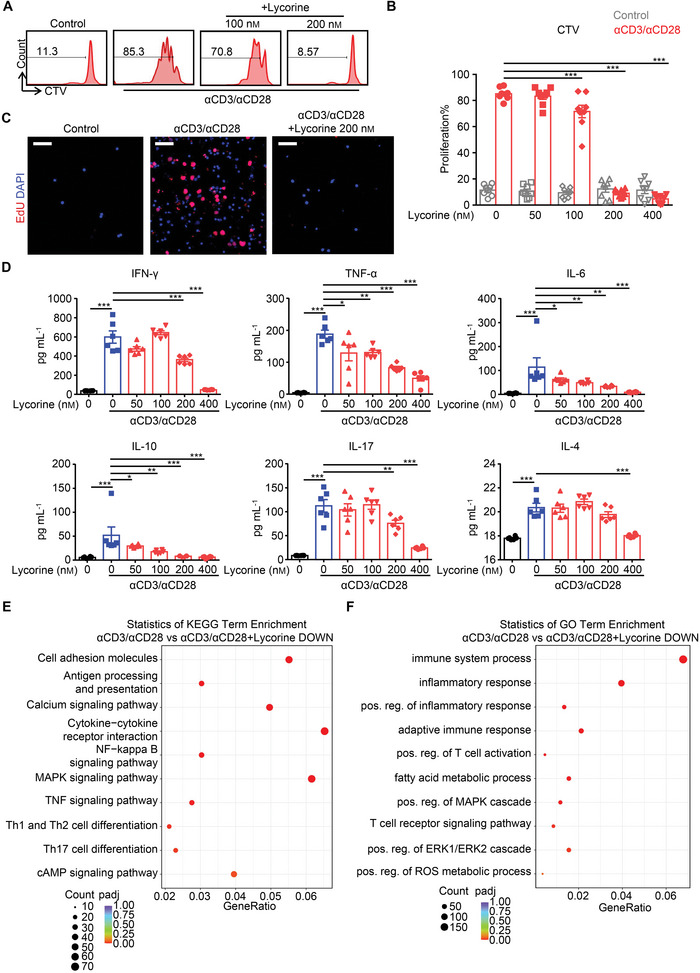
Lycorine inhibits mouse T‐cell proliferation, cytokine production, and immune reaction in vitro. Unsorted splenocytes or purified CD4^+^ T cells from C57BL/6 mice were stimulated with anti‐CD3/anti‐CD28 beads in the presence/absence of lycorine (200 nm or 400 nm). After 72 h, the cells were collected and analyzed. A,B) Purified CD4^+^ T cells were labeled with CellTrace Violet (CTV) dye before stimulation. Cell proliferation was measured by a CTV‐dilution assay. A) Representative histogram and B) bar graph showing percentages of divided cells in each group (*n* = 8). C) Splenocytes were stimulated for 48 h and then 5‐ethynyl‐2′‐deoxyuridine (EdU) was added. At 72 h, immunofluorescent staining for EdU was performed. Representative images of EdU staining in splenocytes without stimulation (Control), with anti‐CD3/anti‐CD28 stimulation (*α*CD3/*α*CD28), or with stimulation in the presence of lycorine (*α*CD3/*α*CD28+Lycorine 200 nm). Scale bars, 50 µm. D) Cytokine production in culture supernatant from purified CD4^+^ T cells receiving the various treatments was detected by CBA assay. Bar graphs show the concentrations of IFN‐*γ*, TNF‐*α*, IL‐6, IL‐10, IL‐17, and IL‐4 in the various treatment groups (*n* = 6 per group). E,F) RNA‐Seq analysis of for purified CD4^+^ T cells with anti‐CD3/anti‐CD28 stimulation or with anti‐CD3/anti‐CD28 stimulation in the presence of lycorine (200 nm). Scatter plots showing E) the Kyoto Encyclopedia of Genes and Genomes (KEGG) enrichment and F) GO enrichment results in the downregulated genes. The dot size indicates the relative number of differentially expressed genes and the shade of the dots indicates the adjusted *P* value (padj) of the enrichment. Data are shown as means ± SEM of at least three independent experiments. *P < 0.05, **P < 0.01, and ***P < 0.001, one‐way ANOVA.

Given the inhibitory effects of lycorine on ERK phosphorylation and allograft rejection, we examined the effects of lycorine on TCR‐dependent T cell proliferation and cytokine production in vitro. To this end, we used beads similar in size to antigen‐presenting cells that had been covalently coupled to anti‐CD3 and anti‐CD28 antibodies to stimulate purified CD4^+^ T cells in vitro. Control T cells proliferated significantly in response to anti‐CD3/anti‐CD28 stimulation, whereas lycorine treatment inhibited this proliferation in a dose‐dependent manner. When the concentration of lycorine reached 200 nm, the proliferation of T cells stimulated by the beads was almost completely suppressed (**Figure** **5**A,B). Immunofluorescent staining with 5‐ethynyl‐2′‐deoxyuridine (EdU) in splenocytes further confirmed the inhibitory effect of lycorine on T‐cell proliferation. EdU‐positive cells were extensively increased in the anti‐CD3/anti‐CD28‐stimulated control group, but they almost completely disappeared in the lycorine‐treated (200 nm) group (Figure 5C).

We then measured cytokine levels in cell culture supernatants using a cytokine bead array approach. We found that the levels of IFN‐*γ*, TNF‐*α*, IL‐6, IL‐10, and IL‐17 increased significantly in response to anti‐CD3/anti‐CD28 stimulation but were dramatically reduced in the presence of lycorine at 200 or 400 nm (Figure 5D). IL‐4 levels were also markedly increased in the control group after stimulation, but this increase was only significantly inhibited in the group treated with 400 nm lycorine. To examine the transcriptomic changes that had occurred in T cells in response to lycorine, we perform RNA‐seq on anti‐CD3/anti‐CD28‐treated CD4^+^ T cells in the presence/absence of lycorine (200 nm). We found that genes in categories such as the calcium signaling pathway, T‐cell differentiation, and the MAPK signaling pathway that were enriched in a Kyoto Encyclopedia of Genes and Genomes (KEGG) analysis, were downregulated in the group treated with anti‐CD3/anti‐CD28+lycorine (Figure 5E). At the same time, GO analysis found that lycorine treatment downregulated a large number of genes related to immune responses, the MAPK cascade, ERK1/ERK2 cascade, and metabolic processes. These findings indicate that lycorine suppresses TCR‐dependent T‐cell proliferation and cytokine production (Figure 5F).

### 2.6 ERK Inhibition by Lycorine Contributes to Mitochondrial Instability and Leads to T‐Cell Dysfunction in Response to Stimulation

**Figure 6:**
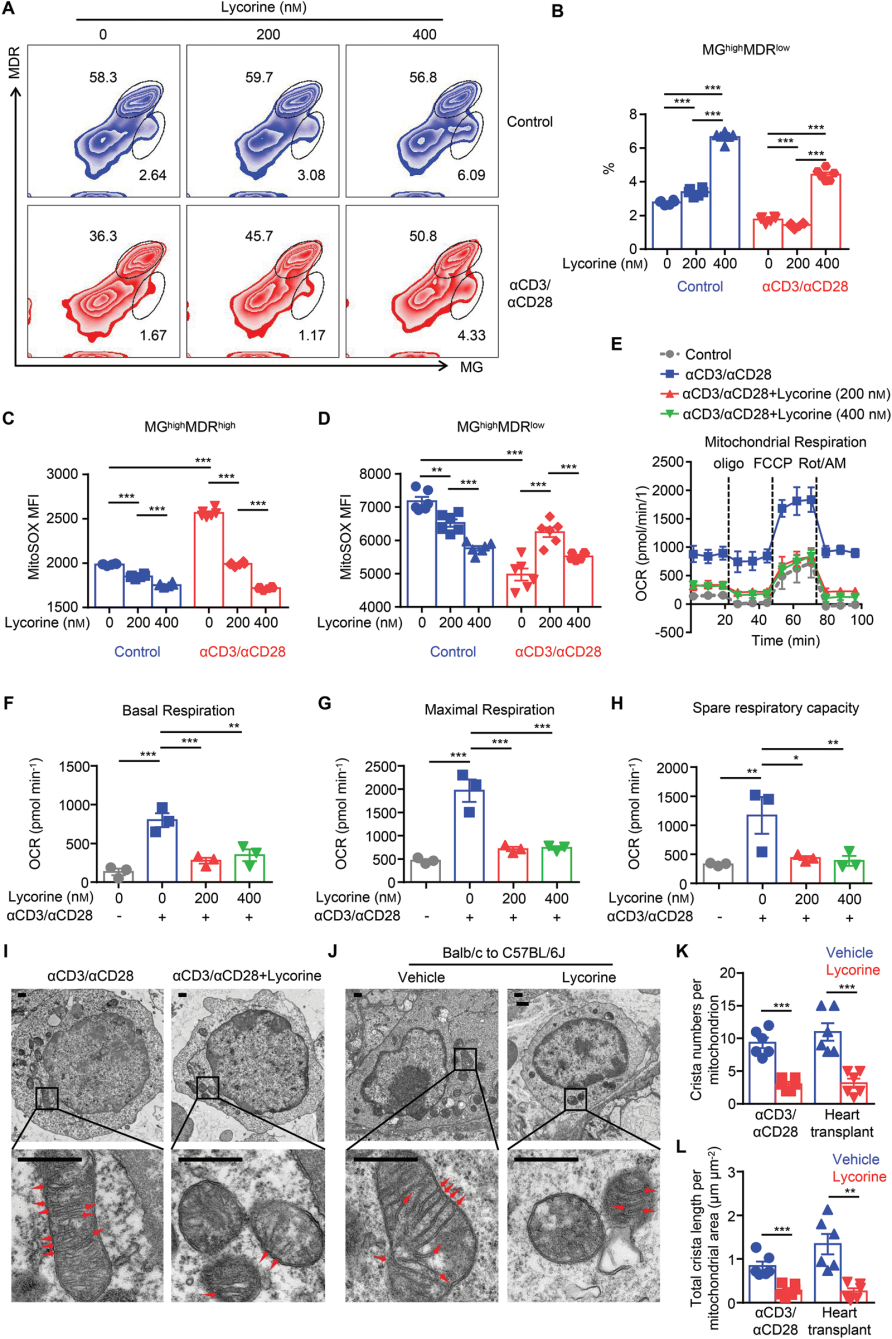
Lycorine treated T cells exhibit mitochondrial dysfunction. A–D) Purified CD4^+^ T cells were stimulated with anti‐CD3/anti‐CD28 beads in the presence/absence of lycorine (200 or 400 nm). After 24 h, cells were collected and stained with MitoTracker Green (MG), MitoTracker Deep Red (MDR), and MitoSOX. A) Representative zebra plot and B) bar graph for the percentage of MG^high^MDR^low^ subsets (*n* = 6). C,D) Summary bar graphs depicting the MFI of MitoSOX in MG^high^MDR^high^ (left panel) and MG^high^MDR^low^ (right panel) subsets in CD4^+^ T cells receiving the various treatments (*n* = 6). E–H) OCR of purified CD4^+^ cells with different treatment were measured under basal conditions and in response to oligomycin (oligo), FCCP, and rotenone plus antimycin A (Rot/AM). E) Real‐time OCR levels, F) baseline OCR levels, G) maximal OCR levels, and H) spare respiratory capacity (percent maximum OCR after FCCP injection of baseline OCR) in the indicated groups (*n* = 3–4 per group). I) Transmission electron microscope sections of mitochondria from CD4^+^ T cells in an in vitro experiment or J) graft‐infiltrating lymphocytes. K) Quantitative plots for crista number per mitochondrion and L) crista length per mitochondrion area in each group (*n* = 6). Scale bars, 500 nm. Bar graphs show the means ± SEM from one of three independent experiments. *P < 0.05, **P < 0.01, and ***P < 0.001, B,F,G,H) one‐way ANOVA, C,D) two‐way ANOVA, and K,L) Student's *t‐*test.

Recent advances have highlighted the observation that a dysfunctional status of T cells, characterized by impaired effector functions and low metabolic activity, is associated with diminished ERK activation in tumors.^[^ ^19^ ^]^ Given the close relationship between the ERK pathway and cell metabolism, we asked whether ERK inhibition by lycorine would interfere with T‐cell metabolism. For this purpose, we first analyzed the mitochondrial mass, mitochondrial membrane potential (MMP), and mitochondrial reactive oxygen species (mtROS) in CD4^+^ T cells after various treatments in vitro by staining with MitoTracker Green (MG), MitoTracker Deep Red (MDR), and MitoSOX, respectively. T cells showing higher MG staining but a lower MDR signal (referred to as MG^high^MDR^low^) have been reported to be depolarized and dysfunctional, whereas T cells with higher MDR signaling (MG^high^MDR^high^) are considered to have relative normal function.^[^ ^20^ ^]^ Here, we found that the percentage of MG^high^MDR^low^ T cells was significantly increased after lycorine (400 nm) treatment, regardless of whether the cells were stimulated with anti‐CD3/anti‐CD28 or not (**Figure** **6**A,B). The dynamic changes we saw in MitoSOX further confirmed the dysfunctional state of the mitochondria in the T cells after lycorine treatment. The MG^high^MDR^high^ T cells showed an increased MitoSOX level in response to anti‐CD3/anti‐CD28 stimulation, whereas this increase was significantly eliminated by simultaneous lycorine addition (Figure 6C). MG^high^MDR^low^ T cells showed high levels of MitoSOX before treatment, but the levels were significantly reduced in response to anti‐CD3/anti‐CD28 stimulation (Figure 6D). Interestingly, we observed that T cells treated with another reported ERK inhibitor, SCH772984, also increased the percentage of MG^high^MDR^low^ subset, and decreased MitoSOX level of MG^high^MDR^high^ subset in response to anti‐CD3/anti‐CD28 stimulation (Figure S4A–C, Supporting Information). Therefore, we conclude that ERK inhibition by lycorine resulted in T‐cell dysfunction, probably because of the elimination of mtROS production in functional T cells and the induction of mitochondrial abnormalities in the T cells.

Given that mtROS is crucial for T‐cell signaling and metabolism,^[^ ^21^ ^]^ we further compared the mitochondrial oxygen consumption rates (OCR) of CD4^+^ T cells with and without anti‐CD3/anti‐CD28 stimulation and in the presence/absence of lycorine (200 or 400 nm). We found that T cells stimulated with anti‐CD3/anti‐CD28 had higher basal and maximal OCR, and these increased levels were significantly reduced by the simultaneous addition of lycorine (200 or 400 nm) (Figure 6E–G). At the same time, a markedly reduced mitochondrial spare respiratory capacity (SRC) was also found in the anti‐CD3/anti‐CD28‐stimulated T cells that had been treated with lycorine (200 or 400 nm), as indicated by a lower increase in OCR following uncoupling with flurocarbonyl cyanide phenylhydrazone (FCCP) (Figure 6H). In addition, we examined the mitochondrial ultrastructure of CD4^+^ T cells under the electron microscope. The mitochondria in lycorine‐treated T cells were disrupted, with fewer cristae, which were also shorter in length (Figure 6I,K,L). In line with these findings, graft‐infiltrating lymphocytes from lycorine‐treated transplant recipients exhibited significant phenotypic damage, with disrupted membrane structures including cristae that were shortened and decreased in number (Figure 6J–L).

### 2.7 Lycorine‐Treated Human Peripheral Blood Mononuclear Cells (PBMCs) Show Hyporesponsiveness

**Figure 7:**
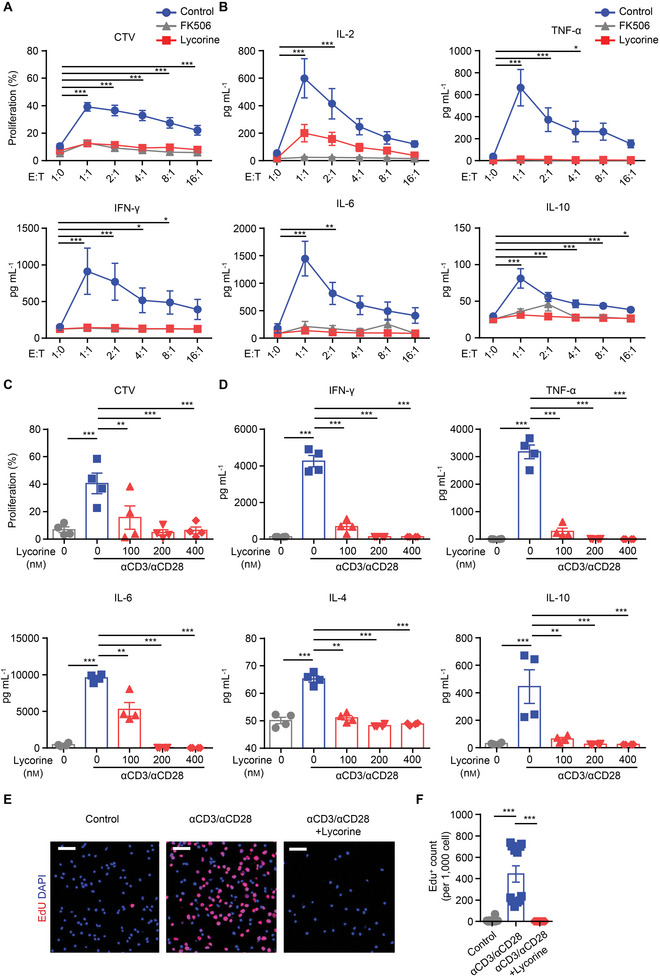
Lycorine treatment inhibits human T‐cells reactivity in response to stimulus. Peripheral blood mononuclear cells (PBMCs) from healthy donors were isolated by density gradient centrifugation. A,B) A mixed lymphocyte reaction was performed at various mixing ratios, and cell proliferation and cytokine levels were measured on day 6 and day 3, respectively. Lycorine (200 nm) or FK506 (200 nm) was added simultaneously with allogenic PBMCs. A) Percentages of proliferated PBMCs and B) levels of cytokines in the culture supernatant from the various groups are shown (*n* = 9). C,D) Dynabeads Human T‐Activator CD3/anti‐CD28 were used for stimulating PBMCs. After 72 h, C) PBMC proliferation and D) cytokine production were analyzed by flow cytometry (*n* = 4). E) Representative images of 5‐ethynyl‐2′‐deoxyuridine (EdU) staining and F) numbers of cells positively stained for EdU (per 1000 cell) in PBMCs without stimulation (Control), with anti‐CD3/anti‐CD28 stimulation (*α*CD3/*α*CD28), or with stimulation in the presence of lycorine (200 nm) (*α*CD3/*α*CD28+Lycorine). Scale bars, 50 µm. Data are shown as means ± SEM and pooled from at least three independent experiments. *P < 0.05, **P < 0.01, and ***P < 0.001, one‐way ANOVA.

Many natural products exhibit remarkable activities in mice but are less effective in humans. In order to comprehensively evaluate the immunosuppressive activity of lycorine, we tested the inhibitory effects of lycorine on human PBMCs with exposed to various stimuli. Using a mixed lymphocyte reaction (MLR) system, we found that a strong proliferation coincided with the pro‐inflammatory cytokine production by T cells in response to allogeneic PBMC stimulation; this proliferation was dramatically abrogated in the presence of lycorine (**Figure** **7**A,B). These inhibitory effects were comparable to those in PBMCs treated with FK506 (tacrolimus) (Figure 7A,B). In addition, anti‐CD3/anti‐CD28 stimulation induced an intense pan‐T‐cell proliferation and Th1/Th2 cytokine secretion, which was significantly attenuated by lycorine treatment (Figure 7C–F).

**Figure 8:**
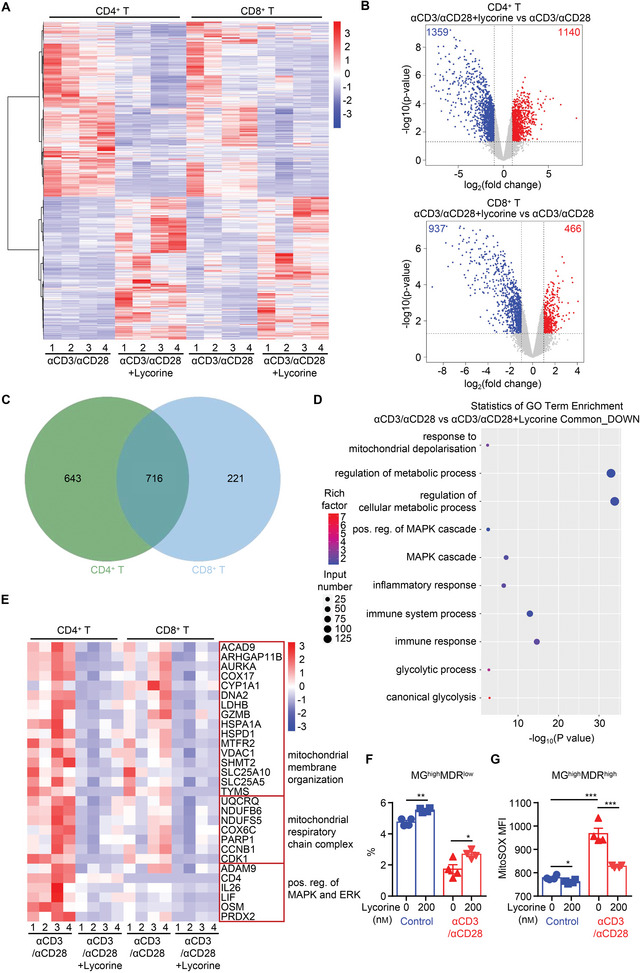
Lycorine‐mediated inhibition of ERK in human T cells downregulates the immune response and changes metabolic processes occurring in response to stimulation. CD4^+^ and CD8^+^ T cells were purified from four healthy individuals and stimulated with anti‐CD3/anti‐CD28 beads (*α*CD3/*α*CD28) or anti‐CD3/anti‐CD28 beads in the presence of lycorine (200 nm) (*α*CD3/*α*CD28+Lycorine). A) Clustered heatmap of significantly differentially expressed mRNAs in CD4^+^/CD8^+^ T cells receiving various treatments. B) Volcano plots indicate the significance of upregulated genes (red) and downregulated genes (blue) in *α*CD3/*α*CD28 and *α*CD3/*α*CD28+Lycorine‐treated CD4^+^ (upper)/CD8^+^ (bottom) T cells, with the number of differentially expressed genes. C) Venn diagram showing the overlapping of the downregulated mRNAs in CD4^+^ and CD8^+^ T cells after lycorine treatment. D) Scatter plot showing the GO enrichment results in the downregulated genes. The dot size indicates the relative number of differentially expressed genes contained in the GO terms, and the shade of the dots indicates the adjusted *P*‐value (padj) of the enrichment. E) Heat maps show standardized log expression (gene‐wise Z‐score) of selected DEGs between CD4^+^ and CD8^+^ T cells stimulated with anti‐CD3/anti‐CD28 + Lycorine versus CD4^+^ and CD8^+^ T cells stimulated with anti‐CD3/anti‐CD28. F,G) Human PBMCs were stimulated with anti‐CD3/anti‐CD28 beads in the presence/absence of lycorine (200 nm). After 24 h, cells were collected and stained with MG, MDR, and MitoSOX. F) Bar graph for the percentage of MG^high^MDR^low^ subsets (*n* = 4). G) Summary bar graphs depicting the MFI of MitoSOX in MG^high^MDR^high^ subsets in CD3^+^ T cells receiving the various treatments (*n* = 4). Data are shown as means ± SEM and from one of three independent experiments. *P < 0.05, **P < 0.01, and ***P < 0.001, Student's *t‐*test.

We then purified CD4^+^ and CD8^+^ T cells from PBMCs obtained from four healthy volunteers, stimulated the T cells with anti‐CD3/anti‐CD28 in the presence/absence of lycorine, and cells were collected 72 h later for RNA‐seq analysis. As shown in **Figure** **8**A, the trends in terms of upregulation and downregulation of genes in response to lycorine treatment were similar between CD4^+^ and CD8^+^ T‐cell groups. According to the values for fragments per kilobase of exon per million mapped reads (FRKM), 1359 and 937 transcripts at a ‐fold change >1 and a false discovery rate (FDR) of <0.05 were downregulated in the lycorine‐treated CD4^+^ and CD8^+^ T cells, respectively, when compared to the corresponding control values (Figure 8B). When we analyzed the commonly downregulated genes in lycorine‐treated cells by using GO enrichment analysis, we identified a series of genes related to immune responses, mitochondrial depolarization, metabolic processes, and the MAPK cascade (Figure 8C,D). GSEA analysis confirmed that lycorine‐treated CD4^+^ and CD8^+^ T cells showed upregulated genes enriched in negative modulation of MAPK and ERK signaling pathways. Meanwhile, mitochondrial function, oxidative phosphorylation (OXPHOS) function and immune response were significantly downregulated after lycorine treatment (Figure S5, Supporting Information). Further analysis of key genes involved in critical regulation of these processes revealed that the genes related to mitochondrial intima protein transporter, mitochondrial electron transport chain complex, and MAPK and ERK pathways were significantly downregulated after lycorine treatment (Figure 8E). Similar to those observed in mice, human T cells treated with lycorine increased the percentage of MG^high^MDR^low^ subset, and decreased MitoSOX level of MG^high^MDR^high^ subset in response to anti‐CD3/anti‐CD28 stimulation (Figure 8F,G). Collectively, these data indicate that lycorine can suppress inflammatory responses of human PBMCs to stimuli, suggesting that it has prominent immunosuppressive effects in human beings and thus potential for clinical application.

## 3 Discussion

During T cell activation, an early wave of protein tyrosine kinase activity begins once allorecognition and costimulatory receptor engagement have occurred.^[^ ^22^ ^]^ Downstream signaling events include the activation of Ras and Rho‐family GTPases, MAPK cascades, and the phosphatidylinositol 3 kinase, PKC*θ*, Jak‐signal transducer and activator of transcription (STAT), and NF‐*κ*B pathways.^[^ ^23^ ^]^ At the same time, TCR ligation leads to phosphorylation and activation of PLC‐*γ*1, which initiates inositol phospholipid turnover and intracellular free ionized calcium flux.^[^ ^24^ ^]^ Although activation of the MAPK pathway has been observed during T‐cell responses,^[^ ^25^ ^]^ little attention has been paid to the dynamics of the ERK pathway in alloreactive T cells or the role of ERK in organ transplantation and allograft rejection.

In the present study, GO analysis showed some upregulated genes in activated alloreactive T cells were enriched in the ERK pathway, and for the first time we observed elevated phosphorylation of ERK1/2 and downstream c‐Fos in infiltrating T cells in rejected allografts. All the evidence points to the conclusion that the ERK pathway is activated in alloreactive T cells, implying that ERK may be a therapeutic target for allograft rejection.

To date, few pharmacological substances specifically targeting the ERK protein have been reported to be effective in preventing allograft rejection. As reported previously,^[^ ^26^ ^]^ the ERK signaling cascade contains Ras‐Raf‐MEK‐ERK signal transduction. Although PD98059 was previously reported to prolong cardiac allograft survival by inhibiting ERK1/2 signaling, it has been reported elsewhere that this inhibitor binds to the inactive form of MEK, thereby preventing upstream kinases from activating MEK1/2, which in turn affects downstream ERK activation.^[^ ^14^, ^27^ ^]^ We have now identified lycorine, an amaryllidaceae alkaloid extracted from the traditional Chinese medicine *Zephyranthes candida*, as a novel specific inhibitor of ERK1 (MAPK3), confirming the direct interaction between lycorine and ERK1 by both the SPR technology and the pull‐down assays. Virtual docking revealed that lycorine may alter the conformation of ERK and lead to the loss of ERK activity. Our in vitro experiments further established that the phosphorylation of ERK and downstream c‐Fos induced by T‐cell activation was significantly inhibited in the presence of lycorine. In contrast, lycorine treatment showed little effects on suppressing MEK1/2 phosphorylation. These results support lycorine as a specific small‐molecule inhibitor of ERK.

To verify that ERK‐specific inhibition can exert a strong immunosuppressive effect in vivo, we tested lycorine in a stringent mouse cardiac allotransplant model (Balb/c‐to‐C57BL/6). We observed that lycorine monotherapy significantly prolonged cardiac allograft survival (median survival time (MST), 24 d), and was even more effective than tacrolimus monotherapy (MST, 12 d). In addition, lycorine used alone was more efficient than PD98059 (MST, 12 d) or cyclosporine A alone (MST, 15 d), as reported in the literature in the same murine model.^[^ ^14c^ ^]^ These results indicate that ERK signaling plays an important role in the development of allograft rejection. ERK‐specific inhibitors have the potential for use as immunosuppressive agents, but given the moderate affinity of lycorine for ERK1, additional congeners and derivatives of lycorine are now beginning to be investigated for their role in inhibiting ERK signaling.

It has been reported that lycorine possessed varied biologic activity, including antifibrosis,^[^ ^28^ ^]^ organ protection,^[^ ^29^ ^]^ and anticancer activity.^[^ ^30^ ^]^ The ERK inhibitory capacity of lycorine might partially involve in these activities because ERK signaling has been reported related to fibrosis,^[^ ^31^ ^]^ organ dysfunction,^[^ ^32^ ^]^ and cancer cell proliferation and apoptosis.^[^ ^33^ ^]^ Compared with other reported synthesized ERK inhibitors (SCH772984, ASTX029, and JSI287),^[^ ^34^ ^]^ the herb‐derived lycorine has a smaller molecular weight and may have advantages of lower extraction cost, higher tissue permeability, higher stability, and less immunogenicity. In addition, only lycorine has been shown to be able to inhibit allograft rejection. However, as a natural product, lycorine may have other unknown effects besides its specific inhibitory effect on ERK. Moreover, lycorine was found to be cleared relatively quickly (less than 2 h) after administration,^[^ ^35^ ^]^ which may not be conducive to maintaining its sustained pharmacological effects in vivo. Previous studies have reported that lycorine has trivial toxicity to normal cells and normal bodies.^[^ ^36^ ^]^ In the present study, we also did not observe any acute and cumulative toxicity of lycorine at the dose we used (5 mg kg^−1^, q.d. or b.i.d.).

ERK substrates have been reported to be involved in the regulation of six fundamental cellular processes: cell proliferation, survival, growth, metabolism, migration, and differentiation.^[^ ^37^ ^]^ Previous study has reported a protective role for ERK inhibition in ischemia‐reperfusion injury.^[^ ^38^ ^]^ However, little is known about the role of ERK in regulating T‐cell metabolism. An emerging theme in immunology is the close relationship between cellular metabolism and T‐cell function, as metabolic reprogramming and lymphocyte activation are intricately linked.^[^ ^39^ ^]^ Although rapidly proliferating effector T cells do prefer glycolysis,^[^ ^40^ ^]^ it is important to note that OXPHOS still undergoes oxygen consumption by these cells.^[^ ^41^ ^]^ In this study, we found that T cells increased their oxygen consumption in response to stimulation, and ERK inhibition by lycorine almost completely eliminated this elevated oxygen consumption. The remaining oxygen consumption capacity of lycorine‐treated T cells indicated that the inhibitory effect was not the result of drug cytotoxicity. A series of categories of genes related to cell metabolic change were found to be enriched in our RNA‐seq experiments comparing lycorine‐treated and untreated T cells. This evidence suggests that ERK inhibition reprograms T‐cell metabolic pathways and leads to T‐cell dysfunction.

Mitochondria act as the “powerhouses” of the cell and are responsible for producing most of its adenosine triphosphate (ATP) under aerobic conditions.^[^ ^42^ ^]^ Mitochondrial fitness is a key factor in determining the fate of T cells in terms of function/dysfunction.^[^ ^20^, ^43^ ^]^ Activated T cells have been reported to show an increase in overall mitochondrial function, including mitochondrial mass, mitochondrial membrane potential, and mitochondrial ROS, consistent with condensed cristae, in response to metabolic demands.^[^ ^44^ ^]^ In contrast, dysfunctional T cells display aberrant mitochondrial function and abnormal mitochondrial morphology. In this study, we found that lycorine treatment induced more dysfunction in T cells, as evidenced by the lower numbers of mitochondria and the less‐condensed cristae found in T cells treated with lycorine in vitro or in vivo. Thus, we contend that ERK inhibition by lycorine can cause mitochondrial dysfunction, alter T cell metabolism, and lead to a reduced T‐cell responsiveness to stimulation.

## 4 Conclusion

In conclusion, our study highlights an association between ERK activation in T cells and allograft rejection and demonstrates that the lycorine therapy we describe here, which specifically targets the ERK pathway, can achieve remarkable results in suppressing T‐cell activation and prolonging allograft survival. Mechanistically, we show for the first time that T cells treated with this ERK inhibitor suffer from mitochondrial dysfunction, leading to metabolic reprogramming in response to stimulation. These findings reveal the close relationship between ERK signaling and allograft rejection and provide the experimental basis for the development of immunosuppressive agents targeting the ERK pathway.

## 5 Experimental Section

### Mice

Specific pathogen‐free C57BL/6J and Balb/c mice were purchased from Charles River Laboratories (Beijing, China). Mice were maintained under controlled conditions (22 °C, 50% humidity, 12 h light/dark cycle, with lights on at 7:00 AM).

### Human Samples

Blood samples were collected from healthy donors at the Wuhan Blood Center. PBMCs were isolated from donors by Ficoll density gradient centrifugation.

### Reagents

Here, the lycorine was obtained in part from the *Zephyranthes candida* but was also purchased from Yuquan Biotechnology Co., Ltd, Xi'an, Shanxi Province, People's Republic of China. The purity of the lycorine (>95%) was determined via high performance liquid chromatography (HPLC) with a UV detector. The preparation of biotinylated lycorine is described in the Supporting Information. Advanced molecular docking between lycorine and ERK was analyzed by using Discovery Studio. SCH772984 was purchased from MedChemExpress (MCE).

### SPR Spectroscopy, Protein Pull‐Down, and Identification

Recombinant human ERK1 protein (ab116536, Abcam) was bound to Sensor Chip CM5 (Cat: 10315413, GE Healthcare) by using an Amine Coupling Kit (Cat: BR‐1000‐50, GE Healthcare), followed by measuring the association with and dissociation of lycorine from ERK1 through SPR technology (BIAcore). For identification of protein bound to lycorine, a splenocyte lysate was incubated with biotinylated lycorine and passed through a Pierce Spin Column containing streptavidin gel, followed by liquid chromatography(LC‐MS)/MS analysis (EASY‐nLC 1000 for LC and OrbitrapFusion Lumos for MS, ThermoFisher).

### Transplantation Procedures

Heart transplantation in mice was performed according to previously described method.^[^ ^45^ ^]^ In brief, hearts from Balb/c donors were transplanted into 8‐ to 10‐week‐old male C57BL/6 recipient mice. The pulmonary artery and aorta of donor heart were incised, the remaining heart vessels were tied off, and the heart was removed. The anesthetized recipient mouse was opened via a midline incision, and the blood flow of the abdominal aorta and inferior vena cava was interrupted by ligation with 6–0 silk thread. Incisions were made in the recipient's abdominal aorta and inferior vena cava to perform the anastomosis with the donor heart aorta and donor pulmonary artery, respectively. The anastomosis was achieved with 11–0 sutures running continuously; the 6–0 silk thread was removed, and the abdomen was then closed by 5–0 sutures in two layers. Heart graft survival was monitored daily by palpation and the day of complete cessation of heartbeat was considered as the day of rejection. Allografts were harvested for analysis at time of rejection or at the indicated time points.

### Immunoblot Analysis

Cells were homogenized in radio immunoprecipitation assay (RIPA) lysis buffer supplemented with 1% protease inhibitor cocktail and phosphatase inhibitors (KeyGEN BioTECH). Protein extracts were resolved by sodium dodecyl sulfate polyAcrylamide gel electrophoresis (SDS‐PAGE), transferred onto an immunobilon membrane, and analyzed by immunoblotting with the antibodies indicated (**Table** **1**). Horseradish peroxidase‐linked antibody to rabbit immunoglobulin G (ThermoFisher), and horseradish peroxidase‐linked antibody to mouse immunoglobulin G (ThermoFisher) were used as secondary antibodies. Protein expression was detected by chemiluminescence.

**Table 1:** Antibodies and dyes used for western blotting and flow cytometry

Antibodies or reagents	Source	Identifier
T cell signaling antibody sampler kit	CST	cat#:14541T
Zap‐70 (D1C10E) XP rabbit mAb	CST	cat#:3165S; RRID: AB_2218656
PLC*γ*1 (D9H10) XP rabbit mAb	CST	cat#:5690S; RRID: AB_10691383
Phospho‐p38 MAPK (Thr180/Tyr182) (28B10) mouse mAb	CST	cat#:9216S; RRID: AB_331296
p38 MAPK (D13E1) XP rabbit mAb	CST	cat#:8690S; RRID: AB_10999090
SLP‐76 (D1R1A) rabbit mAb	CST	cat#:70896S; RRID: AB_2799792
Phospho‐SAPK/JNK (Thr183/Tyr185) (G9) mouse mAb	CST	cat#:9255S; RRID: AB_2307321
SAPK/JNK antibody	CST	cat#:9252S; RRID: AB_2250373
Phospho‐p44/42 MAPK (Erk1/2) (Thr202/Tyr204) (D13.14.4E) XP rabbit mAb	CST	cat#:4370S; RRID: AB_2315112
p44/42 MAPK (Erk1/2) (137F5) rabbit mAb	CST	cat#:4695S; RRID: AB_390779
Anti‐ERK1/2 antibody [EPR18444]	Abcam	cat#: ab214036;
Beta actin polyclonal antibody	ProteinTech	cat#:20536–1‐AP; RRID: AB_10700003
FITC antimouse CD3 antibody (clone 17A2)	Biolegend	cat#:100204; RRID: AB_312661
APC antimouse CD25 (clone PC61)	Biolegend	cat#:102012; RRID: AB_312861
PE antimouse CD4 (clone GK1.5)	Biolegend	cat#:100408; RRID: AB_312693
Brilliant violet 510 antimouse CD4 (clone GK1.5)	Biolegend	cat#:100449; RRID: AB_2564587
APC/Cyanine7 antimouse CD8a (clone 53–6.7)	Biolegend	cat#:100714; RRID: AB_312753
Brilliant violet 785 antimouse CD45 (clone 30‐F11)	Biolegend	cat#:103149; RRID: AB_2564590
Brilliant violet 650 antimouse NK‐1.1 (clone PK136)	Biolegend	cat#:108736; RRID: AB_2563159
PE antimouse CD19 (clone 1D3/CD19)	Biolegend	cat#:152408; RRID: AB_2629817
APC antimouse CD45 (clone 30‐F11)	Biolegend	cat#:103112; RRID: AB_312977
PE/Cyanine7 antimouse CD45 (clone 30‐F11)	Biolegend	cat#:103114; RRID: AB_312979
FITC Hamster antimouse CD69 (clone H1.2F3)	BD Biosciences	cat#:553236; RRID: AB_396675
Brilliant Violet 421 antimouse CD25 (clone PC61)	Biolegend	cat#:102043; RRID: AB_2562611
PE/Cyanine7 antimouse CD134 (OX‐40) (clone OX‐86)	Biolegend	cat#:119416; RRID: AB_2566155
FITC antimouse CD357 (GITR) (clone DTA‐1)	Biolegend	cat#:126308; RRID: AB_1089125
Brilliant Violet 421 antimouse CD279 (PD‐1) (clone 29F.1A12)	Biolegend	cat#:135221; RRID: AB_2562568
APC antimouse CD366 (Tim‐3) (clone RMT3‐23)	Biolegend	cat#:119706; RRID: AB_2561656
Brilliant Violet 650 antimouse CD223 (LAG‐3) (clone C9B7W)	Biolegend	cat#:125227; RRID: AB_2687209
PE antimouse IFN‐*γ* (clone XMG1.2)	Biolegend	cat#:505808; RRID: AB_315402
FITC antimouse TNF‐*α* (clone MP6‐XT22)	Biolegend	cat#:506304; RRID: AB_315425
APC antihuman IFN‐*γ* (clone 4S.B3)	Biolegend	cat#:502512; RRID: AB_315237
APC/Cyanine7 antihuman CD8 (clone SK1)	Biolegend	cat#:344714; RRID: AB_2044006
Brilliant Violet 510 antihuman CD4 (clone OKT4)	Biolegend	cat#:317444; RRID: AB_2561866
Brilliant Violet 421 antihuman CD3 (clone UCHT1)	Biolegend	cat#:300434; RRID: AB_10962690
Mouse Th1/Th2/Th17 CBA Kit	BD Biosciences	cat#:560485; RRID: AB_2869354
Human Th1/Th2/Th17 CBA Kit	BD Biosciences	cat#:560484; RRID: AB_2869353
FITC antimouse CD107a (LAMP‐1) (clone 1D4B)	Biolegend	cat#:121606; RRID: AB_572007
Phospho‐MEK1/2 (Ser221) (166F8) Rabbit mAb (PE Conjugate)	CST	cat#:16211S; RRID: AB_2798759
Phospho‐p44/42 MAPK (Erk1/2) (Thr202/Tyr204) (E10) Mouse mAb (Alexa Fluor 647 Conjugate)	CST	cat#:4375S; RRID: AB_10706777
Phospho‐c‐Fos (Ser32) (D82C12) XP Rabbit mAb (Alexa Fluor 488 Conjugate)	CST	cat#:8709S; RRID: AB_11179220
CellTrace Violet Cell Proliferation Kit	ThermoFisher	cat#:C34557
MitoTracker Green FM	ThermoFisher	cat#:M7514
MitoTracker Deep Red FM	ThermoFisher	cat#:M22426
MitoSOX Red Mitochondrial Superoxide Indicator	ThermoFisher	cat#:M36008

### Histological Examination and Immunofluorescent Staining

Grafts were fixed in 4% formaldehyde and dehydrated with xylene, absolute ethyl alcohol, and 75% alcohol, then embedded in paraffin wax. Four micron‐thick sections were cut. Changes in the graft histology were monitored by hematoxylin and eosin (H&E) staining. The degree of fibrosis was determined by Masson trichrome staining. T‐cell infiltration and p‐ERK1/2 levels in the grafts were estimated by staining with anti‐CD3 and/or anti‐p‐ERK1/2 primary antibody according to a standard protocol (Servicebio). For EdU staining, cultured cells were treated with EdU (25 µm) for 24 h before harvest. The cells were then washed with PBS containing 3% bovine serum albumin, fixed with 4% paraformaldehyde, permeabilized with 0.4% Triton X‐100, incubated with the EdU staining cocktail, and counterstained with 4',6‐diamidino‐2‐phenylindole (DAPI). Sections were scanned using a Pannoramic MIDI (3D HISTECH).

### Isolation of Allograft‐Infiltrated Cells and Splenocytes

Mice bearing heart allografts were sacrificed on post‐transplant day 7. Grafts were removed and cut into pieces, washed with PBS, and then digested at 37 °C for 30 min in dulbecco's modified eagle medium (DMEM) containing 100 ng mL^−1^ type II collagenase before been pressed through a 70 µm filter. The collected cells were washed, and graft‐infiltrating mononuclear cells were purified by density gradient centrifugation in a 38% Percoll solution (GE Healthcare). Purified splenocytes were prepared by homogenizing with a syringe, followed by passage through a 0.1 mm sterile nylon mesh and density gradient centrifugation in a mouse 1× lymphocyte separation medium (Dakewe).

### Cell Sorting

Murine CD4^+^ T cells or CD8^+^ T cells were sorted by magnetic bead separation. In brief, splenocytes were stained with phycoerythrin (PE)‐labeled antimouse CD4 (Cat: 100408, Biolgend) and allophycocyanin (APC)‐labeled antimouse CD8 (Cat:100712, Biolgend) antibodies, followed by incubation with anti‐APC MicroBeads (Miltenyi). CD8^+^ splenocytes were positively selected by passing these labeled cells through a magnetic field (Miltenyi). CD4^+^ T cells were positively selected by further incubating the eluate with anti‐PE Microbeads (Miltenyi) and passage through an LS column (Miltenyi) in a magnetic field. For detection of mitochondrial statues, naïve CD4^+^ T cells from murine splenocytes were purified by using a Naive CD4^+^ T Cell Isolation Kit (Miltenyi). CD4^+^ and CD8^+^ T cells from human PBMCs were purified by using a CD4^+^ T Cell Isolation Kit and CD8^+^ T Cell Isolation Kit (Miltenyi), respectively.

### In Vitro T‐Cell Stimulation

For murine T cell expansion and activation in vitro, splenocytes or purified T cells were stimulated with Dynabeads Mouse T‐Activator CD3/anti‐CD28 (ThermoFisher). Similarly, human PBMCs or purified CD4^+^/CD8^+^ T cells were stimulated with Dynabeads Human T‐Activator CD3/anti‐CD28 (ThermoFisher) according to the manufacturer's instructions. For MLR, human PBMCs were treated with irradiated allogeneic PBMCs at the ratios indicated. For detection of intracellular cytokine stimulated by PMA/ION/BFA, eBioscience Cell Stimulation Cocktail (plus protein transport inhibitors) (500×) was used for a 4 h stimulation before cellular staining.

### RNA‐Sequencing

Total RNA was extracted from purified T cells with TRIzol (Invitrogen). Total RNAs (2 µg) were used to prepare a stranded RNA sequencing library preparation by the use of a Stranded mRNA Library Prep Kit from DR08502 (Bioyigene) according to the manufacturer's instructions. The library products corresponding to 200–500 bp were enriched, quantified, and finally sequenced on a DNBSEQ‐T7 (mice samples) or Illumina Nova‐Seq 6000 (human samples). The gene expression profiles of the mouse or human T cells exposed to the various treatments were determined by RNA‐Seq data analysis (Bioyigene). In brief, raw sequencing data were first filtered by FastQC; low‐quality reads were discarded, and adaptor sequences were trimmed. After quality filtering, each sample had ≈40.4–54.8 million clean reads. Clean reads from each sample were mapped to the Mus musculus GRCm38 reference genome or Homo sapiens GRCh38 reference genome using the HISAT2 v2.1.0. Significantly differentially expressed transcripts were screened by applying the criteria fold change (FC) ≥2 or ≤ −2 and *P*‐value ≤ 0.05. The RNA‐seq data, entitled “RNA‐seq of T cell with/without lycorine treatment in vitro and RNA‐seq of T cell in spleen and allograft” were deposited in Gene Expression Omnibus (GEO) at GSE216671. GO and KEGG enrichment analysis of differentially expressed genes was conducted using the ClusterProfiler (v3.18.1) software with a padj (FDR) < 0.05 to determine statistically significant enrichment.

### Flow Cytometry

Cells were stained with various fluorochrome‐conjugated antibodies or fluorescent dye, followed by analysis with a BD FACSCelesta cell analyzer (BD Biosciences). Intracellular staining of phosphorylated ERK1/2, MEK1/2, or c‐Fos was performed according to the manufacturer's instructions (Cell Signaling Technology , CST). Intracellular staining of IFN‐*γ* and TNF‐*α* was performed by following the Intracellular Flow Cytometry Staining Protocol (Biolegend). Cytokine levels in mouse serum and culture supernatant were measured with a Cytometric Bead Array (CBA) kit for mouse Th1/Th2/Th17 cytokines or human Th1/Th2/Th17 cytokines according to the manufacturer's instructions. For measuring cell proliferation, cells were stained with CellTrace Violet (CTV), and proliferation was determined by CTV‐dilution assay. Data were analyzed using FlowJo software (TreeStar). Sources and specific information regarding the antibodies, dyes, and kits for flow cytometry used here are listed in Table 1.

### Metabolic Assays

Purified CD4^+^ T cells were suspended in Seahorse XF medium (minimal, unbuffered DMEM supplemented with 2 mm glutamine), and plated on polylysine‐treated Cell‐Tak‐coated Seahorse culture plates (250 000 cells per well) for homogeneous adherence. Cells in different pores were enrolled in one of four groups: an unstimulated group, anti‐CD3/anti‐CD28‐stimulated group, anti‐CD3/anti‐CD28‐stimulated plus lycorine‐treated (200 nm) group, and an anti‐CD3/anti‐CD28‐stimulated plus lycorine‐treated (400 nm) group. The OCR was measured under basal conditions and in response to 1.5 µm oligomycin, 1.5 µm FCCP, and 500 nm rotenone + 500 nm antimycin A (Sigma) using an XF‐24 Extracellular Flux Analyzer (Seahorse Biosciences).

### Statistics

Results are represented as means ± standard error of mean (SEM) and analyzed with Prism software (ver. 6.0, GraphPad). Graft survival was compared using the log‐rank test. Data were analyzed by Student's *t*‐test or paired *t*‐test. The measurement data in multiple groups were compared with one‐way or two‐way analysis of variance (ANOVA). *P*‐values <0.05 were considered statistically significant.

### Study Approval

All animal experiments were conducted according to a protocol approved by Huazhong University of Science and Technology Animal Care and Use Committee. Use of human samples used in this study complied with the Declaration of Helsinki and was approved by the Human Assurance Committee of Tongji Hospital. A statement indicating that written informed consent had been given was obtained from all subjects prior to participation.Chen

## Conflict of Interest

The authors declare no conflict of interest.

## Author Contributions

X.T. and C.Q. contributed equally to this work. G.C., Y.Z., and X.T. conceived and supervised the project. X.T., L.S., X.Z., M.W., H.F., X.H., B.X., P.X., and M.W. performed the in vitro and in vivo experiments. C.Q., W.S., L.G., F.W., and Z.S. performed lycorine extraction and derivatization. X.T., M.W., and C.Q. conducted the data analyses. X.T., G.C., C.Q., and Y.Z. wrote the paper. All authors read and approved the final paper. The order of the cofirst authors was assigned based on the relative contributions of these individuals.

## Supporting information

Supporting Information
Supporting Information is available from the Wiley Online Library or from the author.

## Data Availability

The RNA‐seq data, entitled “RNA‐seq of T cell with/without lycorine treatment in vitro and RNA‐seq of T cell in spleen and allograft” were deposited in Gene Expression Omnibus (GEO) at GSE216671.
