# Supplementary material for: ERK Inhibition Promotes Engraftment of Allografts by Reprogramming T‐Cell Metabolism
Source: Adv Sci (Weinh). 2023 Apr 4;10(16):2206768. doi: 10.1002/advs.202206768 (PMC10238213; doi:10.1002/advs.202206768)
Supplement: Supplementary file 1 — Supporting Information Supporting Information is available from the Wiley Online Library or from the author. [file ADVS-10-2206768-s001.pdf]

ADVANCED SCIENCE
Open Access

Supporting Information

for *Adv. Sci.*, DOI 10.1002/advs.202206768

ERK Inhibition Promotes Engraftment of Allografts by Reprogramming T-Cell Metabolism

Xiaosheng Tan, Changxing Qi, Xiangli Zhao, Lingjuan Sun, Mi Wu, Weiguang Sun, Lianghu Gu, Fengqing Wang, Hao Feng, Xia Huang, Bin Xie, Zhengyi Shi, Peiling Xie, Meng Wu, Yonghui Zhang* and Gang Chen**

Supporting Information

ERK inhibition promotes engraftment of allografts by reprogramming T-cell metabolism

*Xiaosheng Tan**, *Changxing Qi, Xiangli Zhao, Lingjuan Sun, Mi Wu, Weiguang Sun, Lianghu Gu, Fengqing Wang, Hao Feng, Xia Huang, Bin Xie, Zhengyi Shi, Peiling Xie, Meng Wu, Yonghui Zhang**, *Gang Chen**

Figure S1

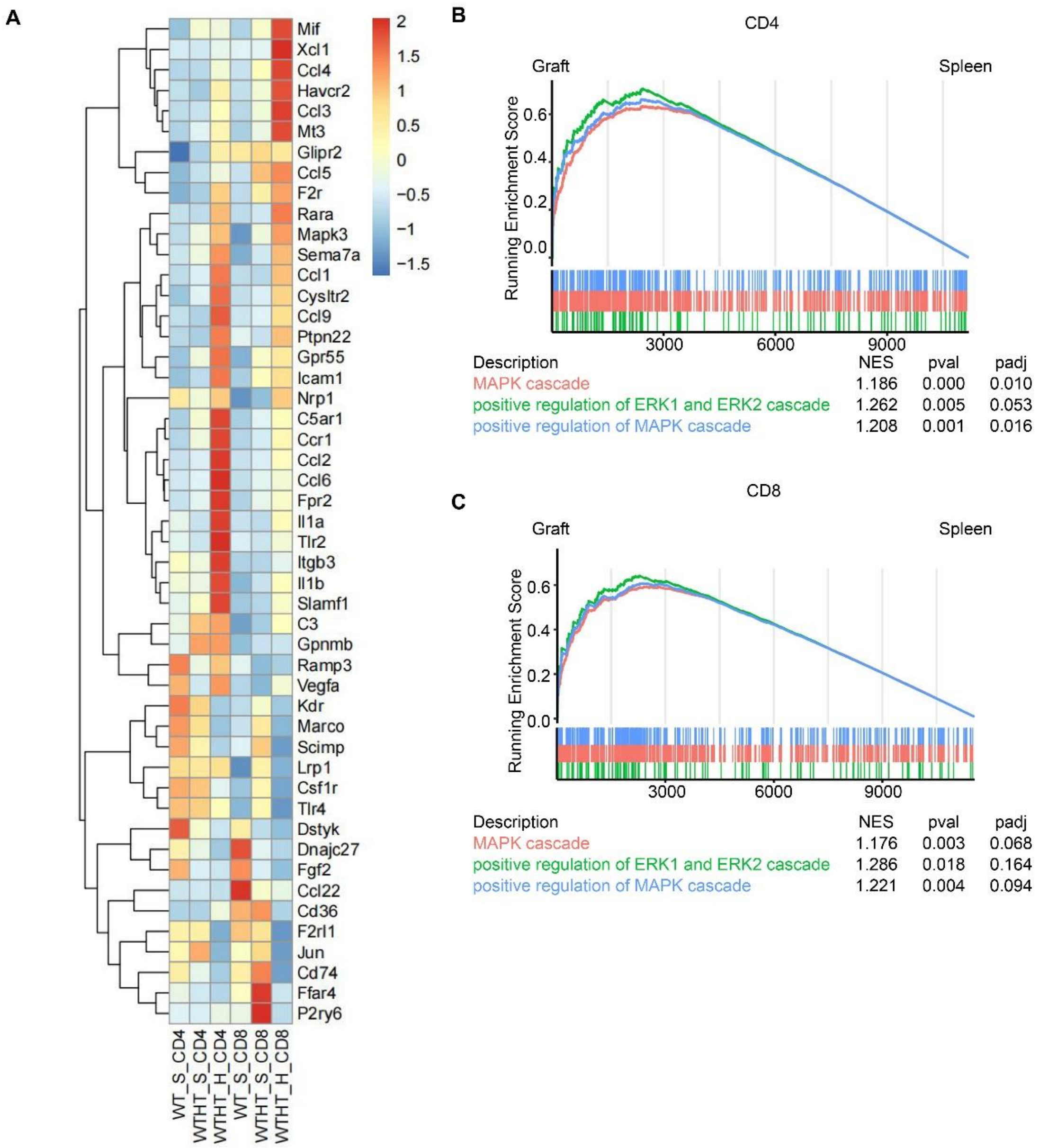

Figure S1. Genes and pathways related to ERK1 and ERK2 cascade in T-cell subsets. A) Clustered heatmap of expressed mRNAs in $CD4^+$ and $CD8^+$ T cells from spleen and grafts. B-C) Gene Set Enrichment Analysis (GSEA) shows an enrichment of ERK1/2- and MAPK-related pathway up- and down-regulated genes in the transcriptome of $CD4^+$ (B) and $CD8^+$

(C) T cells from allograft. WT_S_CD4 and WT_S_CD8: splenic $CD4^+$/$CD8^+$ T cells from mice without transplantation; WTHT_S_CD4 and WTHT_S_CD8: splenic $CD4^+$/$CD8^+$ T cells from mice with allogenic heart transplantation; WTHT_H_CD4 and WTHT_H_CD8: graft-infiltrating $CD4^+$/$CD8^+$ T cells from mice with allogenic heart transplantation.

Figure S2

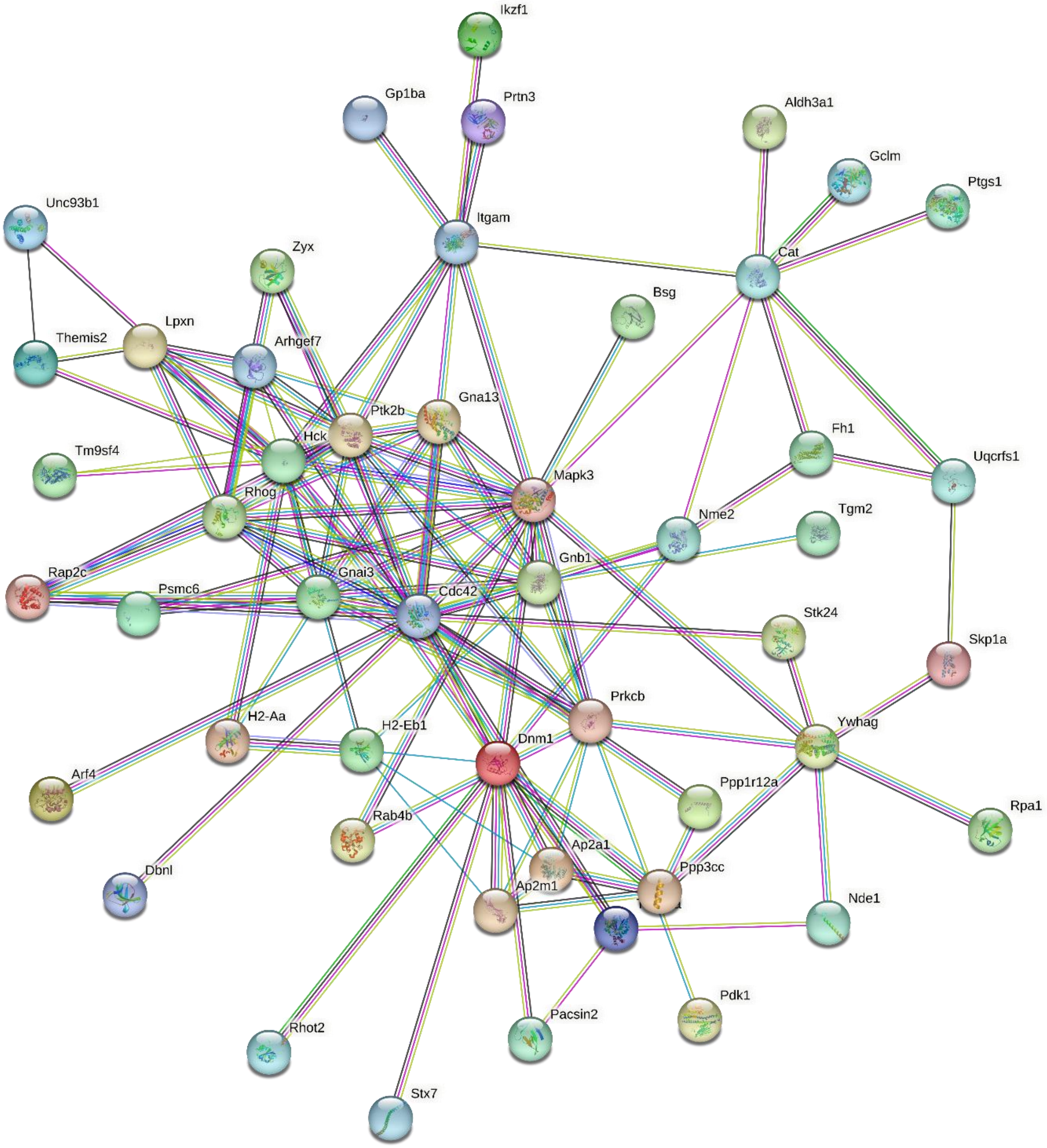

Figure S2. Analysis of the proteins of 194 peptides that can directly or indirectly (<3 connections) interact with ERK1 (Mapk3).

Figure S3

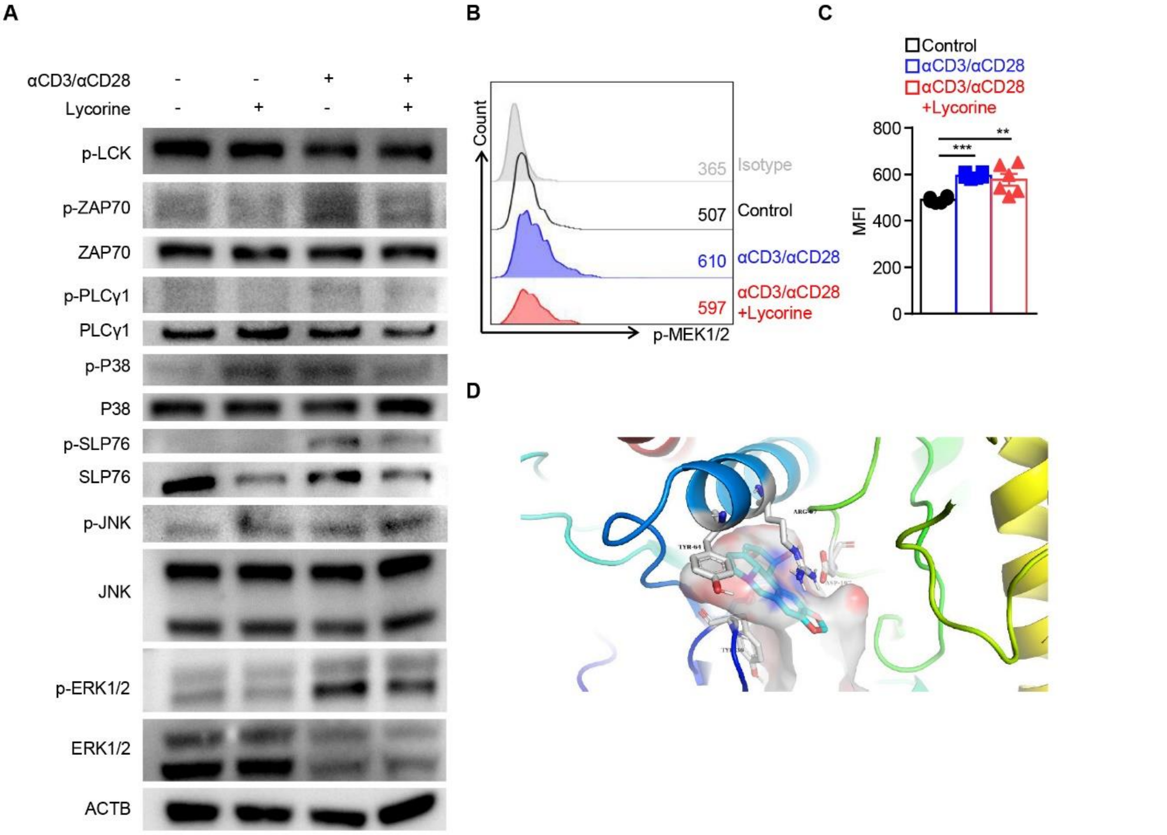

Figure S3. Lycorine treatment reduces ERK1/2 phosphorylation in T cells. A) Mouse CD4$^+$ T cells were purified by magnetic beads, followed by stimulation with Dynabeads™ Mouse T-Activator CD3/anti-CD28 beads (αCD3/αCD28) in the presence/absence of lycorine (200 nM). Cells without stimulation served as a control. After 1 h, phosphorylation levels of proteins in the TCR downstream cascade were measured by western blotting. B-C) Flow cytometry staining for phosphorylated-MEK1/2 (p-MEK1/2) in CD4$^+$ T cells without stimulation, with anti-CD3/anti-CD28 bead stimulation, or with anti-CD3/anti-CD28 beads stimulation in the presence of lycorine (200 nM) for 15 min (n=6 for each group). Representative histogram with mean fluorescence intensity (B) and bar graph with mean ± SEM (C). D) The virtual docking between lycorine and ERK1. Data are shown as means ± SEM and from one of three independent experiments. **P < 0.01, and ***P < 0.001, one-way ANOVA.

Figure S4

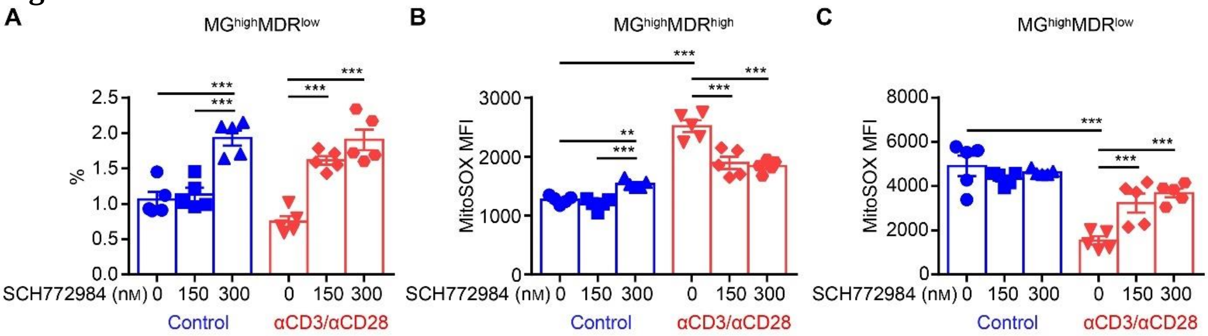

Figure S4. SCH772984 treated T cells exhibit mitochondrial dysfunction. A-C) Purified $CD4^+$ T cells were stimulated with anti-CD3/anti-CD28 beads in the presence/absence of SCH772984 (150 nM or 300 nM). After 24 h, cells were collected and stained with MitoTracker Green (MG), MitoTracker Deep Red (MDR), and MitoSOX. A) Bar graph for the percentage of $MG^{high}MDR^{low}$ subsets (n=5). B-C) Summary bar graphs depicting the MFI of MitoSOX in $MG^{high}MDR^{high}$ (left panel) and $MG^{high}MDR^{low}$ (right panel) subsets in $CD4^+$ T cells receiving the various treatments (n=5). Data are shown as means ± SEM and from one of three independent experiments. **P < 0.01, and ***P < 0.001, one-way ANOVA (A), and two-way ANOVA (B and C).

Figure S5

A

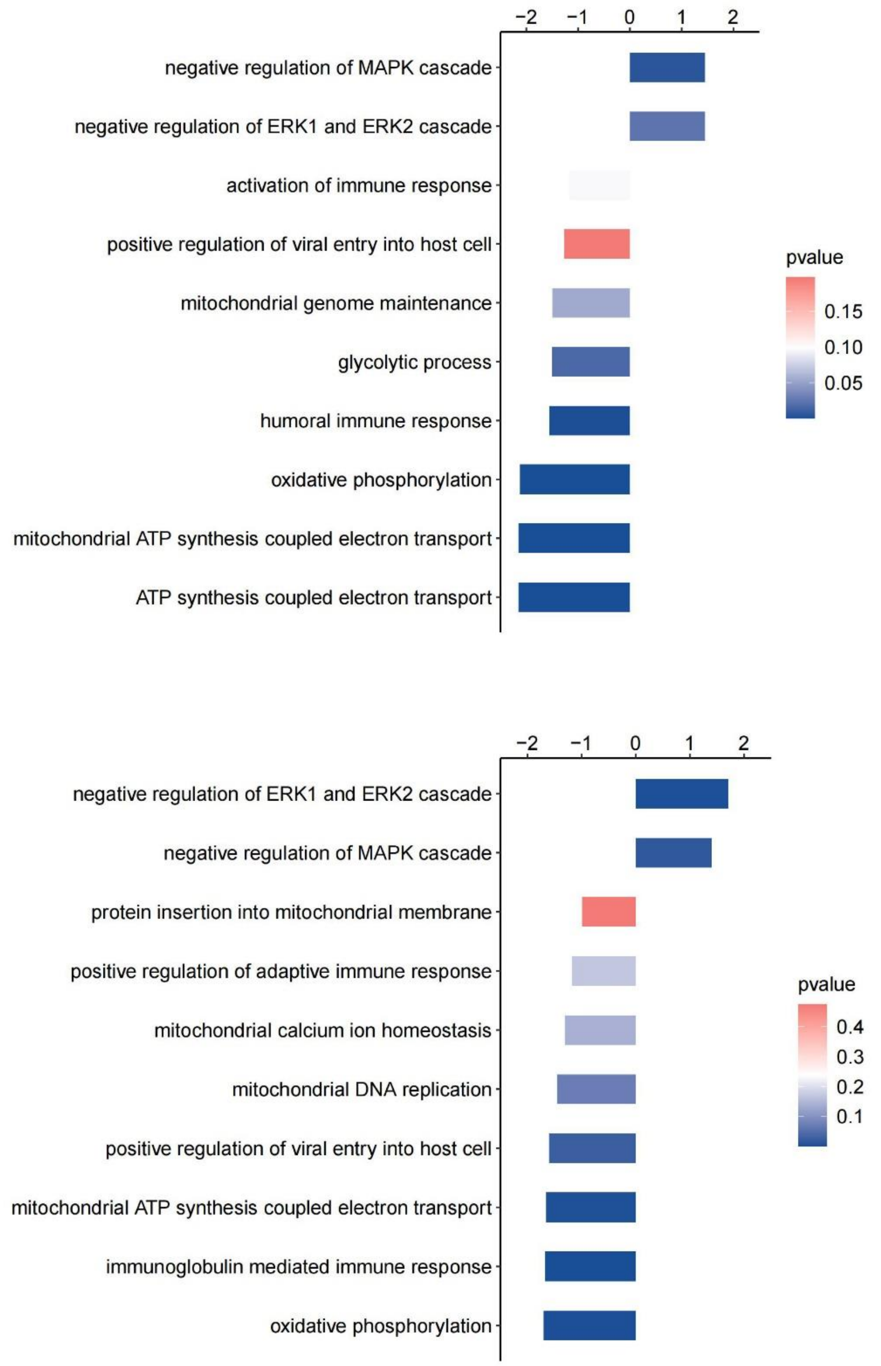

Figure S5. GSEA analysis of enriched gene sets in human T cells with lycorine treatment. Results of GSEA Hallmark analysis showing enriched gene sets from all genes between CD4$^+$ (A) and CD8$^+$ T (B) cell subsets with anti-CD3/anti-CD28 stimulation in the presence/absence of lycorine. Color reflects p-value. A positive normalized enrichment fraction (NES) value indicated enrichment in lycorine treatment group, while negative NES value indicated enrichment in control group.

Supplemental materials

1. Preparation of lycorine

The lycorine in this study was partially obtained from the *Zephyranthes candida* and partially purchased from Yuquan Biotechnology Co., Ltd, Xi'an, Shanxi Province, People's Republic of China.

1.1 Isolation of lycorine

Plant Material. Whole *Zephyranthes candida* plants were collected at Shiyan, Hubei Province, People's Republic of China and identified by Professor Changgong Zhang from Huazhong University of Science and Technology.

Extraction and Isolation. The dried *Z. candida* plants (10 kg) were extracted four times with 25 L each of 95% aqueous EtOH containing 2% HCl at room temperature, and the filtrates were combined and concentrated under vacuum to afford 1150 g of crude extract. Then the concentrated filtrates were partitioned between $CHCl_3$ and 2% aqueous HCl (3 L each), followed by re-extracting the aqueous phase three additional times with $CHCl_3$ (3 L). After the aqueous phase was adjusted to pH 7 with NH_4OH, it was partitioned between $CHCl_3$ (4 × 1.5 L) for a second time. On evaporation, the $CHCl_3$ phase (18 g) was chromatographed over silica gel by MPLC and eluted with a MeOH−$CHCl_3$ gradient to give five fractions, A−E. Fraction E was separated on a RP-C18 column using a MeOH−H_2O gradient to yield three fractions, E1, E2, and E3, and the lycorine (5.4 mg) was found in fraction E2.

1.2 Identification and purity detection of lycorine

By referring to the literature (Evidente, A.; Cicala, M. R.; Giudicianni, I. Phytochemistry, 1983, 22, 581−584), we confirmed that our isolate from *Z. candida* was lycorine. In addition, the isolate's purity was evaluated on an Agilent 1200 system and a Dionex HPLC system with a reversed-phase (RP) C_{18} column (3 µm, 4.6×250 mm, Welch Ultimate AQ-C_{18}) under conditions of 1 mL min^{-1}, MeOH−H_2O−diethylamine = 20:80:0.01 (v:v:v). The results showed that the purity of the used lycorine in this study was >95%.

2. Preparation of biotinylated lycorine (namely WFQ 3-5)

2.1 Synthetic route of biotinylated lycorine (namely WFQ 3-5)

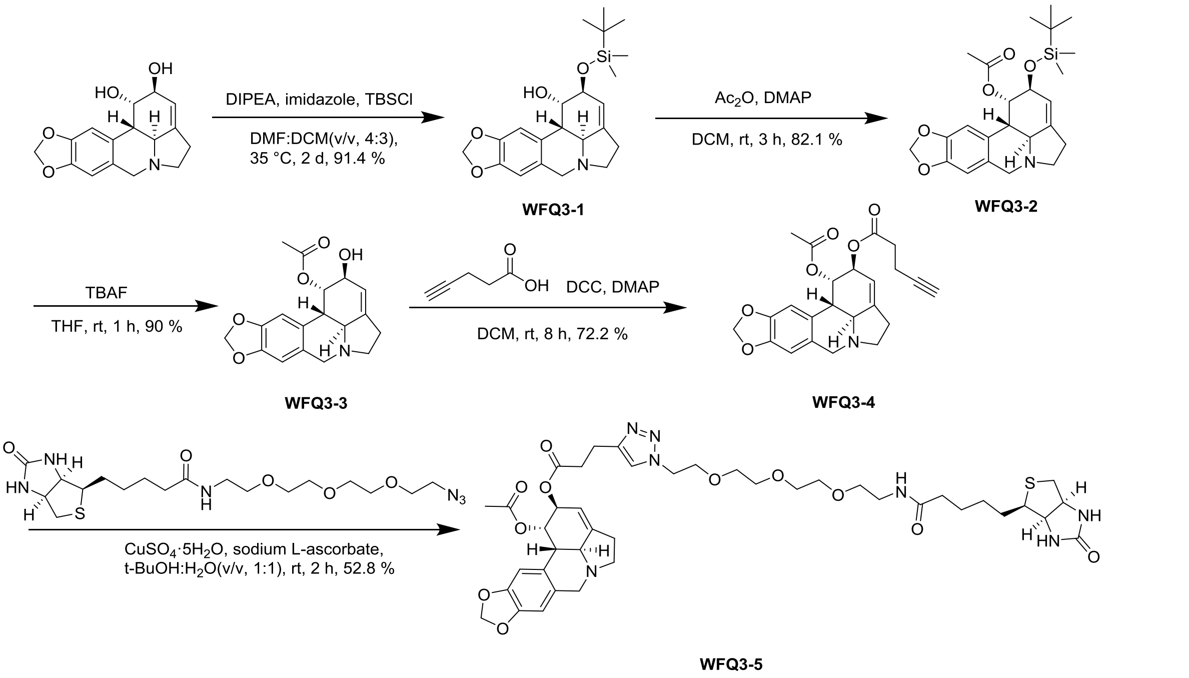

2.2 Preparation of WFQ3-1

To a suspension of lycorine (1 g, 3.5 mmol, 1 eq) in DCM (20 mL), *N,N*-diisopropylethylamine (1.8 mL, 10.5 mmol, 3 eq), imidazole (711 mg, 10.5 mmol, 3 eq), *t*-butyl dimethyl chlorosilane (900 mg, 6 mmol, 1.7 eq), and *N,N*-dimethylformamide (15 mL) were added. The resulting solution was stirred at 35 °C for 2 d. The reaction mixture was then quenched with water (25 mL) and extracted with DCM (3×30 mL). The combined organic layers were washed with brine; dried over Na_2SO_4, and concentrated under reduced pressure. Purification by column chromatography (DCM/MeOH 10/1) afforded **3-1** (1.28 g) as a yellow oil in 91.4% yield.

^{1}H NMR (400 MHz, 25 °C, Methanol-d_4) δ (ppm) 6.80 (s, 1H), 6.62 (s, 1H), 5.89 (q, *J* = 1.2 Hz, 2H), 5.45 (dd, *J* = 2.4, 1.2 Hz, 1H), 4.38 (d, *J* = 2.3 Hz, 1H), 4.25 (dt, *J* = 3.4, 1.7 Hz, 1H), 4.09 (d, *J* = 14.1 Hz, 1H), 3.57 (dt, *J* = 14.2, 1.3 Hz, 1H), 3.31 (dd, *J* = 7.7, 1.8 Hz, 1H), 2.93 (d, *J* = 10.8 Hz, 1H), 2.77 (d, *J* = 10.8 Hz, 1H), 2.70-2.56 (m, 2H), 2.55-2.43 (m, 1H), 0.90 (s, 9H), 0.17 (s, 3H), 0.13 (s, 3H).

^{13}C NMR (101 MHz, 25 ℃, Methanol-d_4) δ (ppm) 148.3, 147.7, 142.2, 129.9, 129.8, 120.2, 108.3, 105.8, 102.4, 73.8, 72.2, 62.4, 57.5, 54.7, 41.2, 29.3, 26.3, 19.0, -4.4, -4.7.

2.3. Preparation of WFQ3-2

Silyl ether **WFQ3-1** (500 mg, 1.24 mmol, 1 eq) was dissolved in DCM (4 mL) at room temperature. 4-Dimethylaminopyridine (304 mg, 2.48 mmol, 2 eq) and Ac_2O (0.18 mL, 1.86 mmol, 1.5 eq) were consecutively added to the solution. The resulting mixture was stirred for 3 h, at which time TLC (DCM/MeOH 15/1) indicated the reaction to be completed. The mixture was then diluted with saturated aqueous sodium bicarbonate (5 mL) and extracted with DCM (5 mL×3). The combined organic layers were washed with brine; dried over Na_2SO_4, and the solvent removed under reduced pressure to yield a gum, which was purified on silica gel and eluted with (DCM/MeOH = 15/1) to give compound **WFQ3-2** (453.3 mg, 82.1%) as a white crystalline powder.

^{1}H NMR (400 MHz, 25 ℃, Methanol-d_4) δ (ppm) 6.66 (s, 1H), 6.64 (s, 1H), 5.91 (q, J = 1.2 Hz, 2H), 5.61 (q, J = 1.7 Hz, 1H), 5.51 (s, 1H), 4.25 (dt, J = 3.5, 1.6 Hz, 1H), 4.16 (d, J = 14.3 Hz, 1H), 3.69 (d, J = 14.3 Hz, 1H), 3.42-3.34 (m, 1H), 3.09-2.94 (m, 2H), 2.78-2.58 (m, 3H), 1.91 (s, 3H), 0.91 (s, 9H), 0.21 (s, 3H), 0.13 (s, 3H).

^{13}C NMR (101 MHz, 25 ℃, Methanol-d_4) δ (ppm) 172.1, 148.6, 148.3, 141.9, 129.6, 128.5, 120.6, 108.7, 105.4, 102.7, 73.3, 71.0, 62.9, 57.2, 54.8, 39.8, 29.5, 26.4, 20.8, 19.0, -4.5.

2.4 Preparation of WFQ3-3

To a solution of silyl acetate **WFQ3-2** (280 mg, 0.63 mmol, 1 eq) in THF (5 mL) at room temperature was added tetrabutylammonium fluoride (TBAF, 1M) solution (0.945 mL, 0.945 mmol, 1.5 eq). The resulting solution was stirred until TLC (DCM/MeOH = 5/1) indicated the reaction to be completed. The mixture was then diluted with saturated NH_4Cl solution (5 mL) and extracted with ethyl acetate (5 mL×3). The combined organic fractions were dried over anhydrous Na_2SO_4, and the solvent was removed under reduced pressure to yield a gum that

was purified on a silica gel column and eluted with (DCM/MeOH = 8/1) to give compound **WFQ3-3** as a light yellow crystalline powder (187.2 mg, 90%).(McNulty et al., 2009)

^{1}H NMR (400 MHz, 25 ℃, Methanol-d_4) δ (ppm) 6.72 (s, 1H), 6.65 (s, 1H), 5.90 (d, J = 1.4 Hz, 2H), 5.69 (q, J = 1.5 Hz, 1H), 5.59-5.54 (m, 1H), 4.17-4.11 (m, 2H), 3.62 (d, J = 14.2 Hz, 1H), 3.38-3.31 (m, 1H), 2.99-2.86 (m, 2H), 2.75-2.61 (m, 2H), 2.61-2.52 (m, 1H), 1.90 (s, 3H).

^{13}C NMR (101 MHz, 25 ℃, Methanol-d_4) δ (ppm) 172.0, 148.3, 148.0, 143.3, 130.0, 128.4, 119.4, 108.4, 105.7, 102.5, 73.3, 70.2, 62.9, 57.4, 54.6, 40.0, 29.3, 20.7.

2.5 Preparation of WFQ3-4

To a solution of acetate **WFQ3-3** (100 mg, 0.3 mmol, 1 eq) in DCM (1 mL) were added 4-pentynoic acid (35.8 mg, 0.36 mmol, 1.2 eq), DMAP (7.42 mg, 0.06 mmol, 0.2 eq), and DCC (75.2 mg, 0.36 mmol, 1.2 eq). The resulting solution was stirred until TLC (DCM/MeOH 10/1) indicated the reaction to be completed. The mixture was diluted with DCM (20 mL) and filtrated through celite. Then the resulting filtrate was successively washed with water and saturated brine. Evaporation and concentration under reduced pressure gave a residue that was further purified by silica gel chromatography (DCM/MeOH = 20/1) to afford **WFQ3-4**, (89.8 mg, 72.2%) as a white solid.

^{1}H NMR (400 MHz, 25 ℃, Methanol-d_4) δ (ppm) 6.74 (s, 1H), 6.65 (s, 1H), 5.91 (d, J = 1.5 Hz, 2H), 5.78 (q, J = 1.5 Hz, 1H), 5.54 (dp, J = 5.1, 2.0 Hz, 1H), 5.30 (dq, J = 3.6, 1.8 Hz, 1H), 4.15 (d, J = 14.3 Hz, 1H), 3.64 -3.55 (m, 1H), 3.37 (ddd, J = 9.6, 7.6, 2.2 Hz, 1H), 2.91 (q, J = 8.1 Hz, 2H), 2.78-2.63 (m, 2H), 2.63-2.44 (m, 5H), 2.27 (t, J = 2.6 Hz, 1H), 1.92 (s, 3H).

^{13}C NMR (101 MHz, 25 ℃, Methanol-d_4) δ (ppm) 172.2, 171.5, 148.2, 148.1, 146.9, 130.3, 127.5, 115.3, 108.4, 105.9, 102.5, 72.1, 70.4, 70.3, 62.7, 57.5, 54.5, 41.4, 34.4, 29.4, 20.6, 15.1, 15.1.

2.6 Preparation of WFQ3-5

To a solution of **WFQ3-4** (20 mg, 0.049 mmol, 1 eq) and azide-PEG3-biotin (21.6 mg, 0.049 mmol,1 eq) in *t*-butanol /H_2O (v/v, 1:1, 0.5 mL) at room temperature were added $CuSO_4 \cdot 5H_2O$ (2.4 mg, 0.010 mmol, 0.2 eq) and sodium L-ascorbate (4 mg, 0.02 mmol, 0.4 eq). The reaction mixture was stirred for 2 h at 25 ºC, and then it was filtered through celite and concentrated under reduced pressure. Purification by column chromatography (DCM/MeOH = 10/1) afforded the biotinylated lycorine, **WFQ3-5**, (22 mg, 52.8 %) as an oil.

^{1}H NMR (400 MHz, 25 ℃, Methanol-d_4) δ (ppm) 7.84 (s, 1H), 6.71 (s, 1H), 6.65 (s, 1H), 5.92 (d, J = 1.6 Hz, 2H), 5.65 (s, 1H), 5.51 (s, 1H), 5.27 (dt, J = 3.6, 1.7 Hz, 1H), 4.54 (t, J = 5.1 Hz, 2H), 4.47 (dd, J = 7.9, 4.8 Hz, 1H), 4.29 (dd, J = 7.9, 4.4 Hz, 1H), 4.15 (d, J = 14.2 Hz, 1H), 3.87 (t, J = 5.1 Hz, 2H), 3.59 (d, J = 8.6 Hz, 8H), 3.51 (t, J = 5.5 Hz, 2H), 3.34 (t, J = 5.5 Hz, 2H), 3.18 (dt, J = 8.9, 5.3 Hz, 1H), 3.03 (t, J = 7.2 Hz, 2H), 2.95-2.88 (m, 2H), 2.83 (d, J = 10.9 Hz, 1H), 2.77 (q, J = 7.0 Hz, 2H), 2.73-2.63 (m, 3H), 2.62-2.45 (m, 2H), 2.19 (t, J = 7.3 Hz, 2H), 1.92 (s, 3H), 1.65 (tdd, J = 30.8, 13.5, 9.5 Hz, 6H), 1.41 (p, J = 7.7 Hz, 2H).

^{13}C NMR (101 MHz, 25 ℃, Methanol-d_4) δ (ppm) 176.1, 172.8, 171.6, 148.1, 148.1, 147.2, 147.0, 131.1, 130.4, 124.4, 115.2, 108.4, 106.0, 102.5, 100.9, 72.1, 71.6, 71.5, 71.4, 71.3, 70.6, 70.6, 70.4, 63.4, 62.6, 61.6, 57.6, 57.0, 54.5, 51.3, 41.4, 41.0, 40.3, 36.7, 34.6, 29.8, 29.5, 29.4, 26.8, 21.9, 20.7.

Reference

[1] J. McNulty, J. J. Nair, M. Singh, D. J. Crankshaw, A. C. Holloway, J. Bastida, Bioorg Med Chem Lett 2009, 19 (12), 3233, https://doi.org/10.1016/j.bmcl.2009.04.086.

^{1}H NMR (400 MHz, 25 □, Methanol-*d*4), ^{13}C NMR (101 MHz, 25 □, Methanol-d_4) for **WFQ3-1**

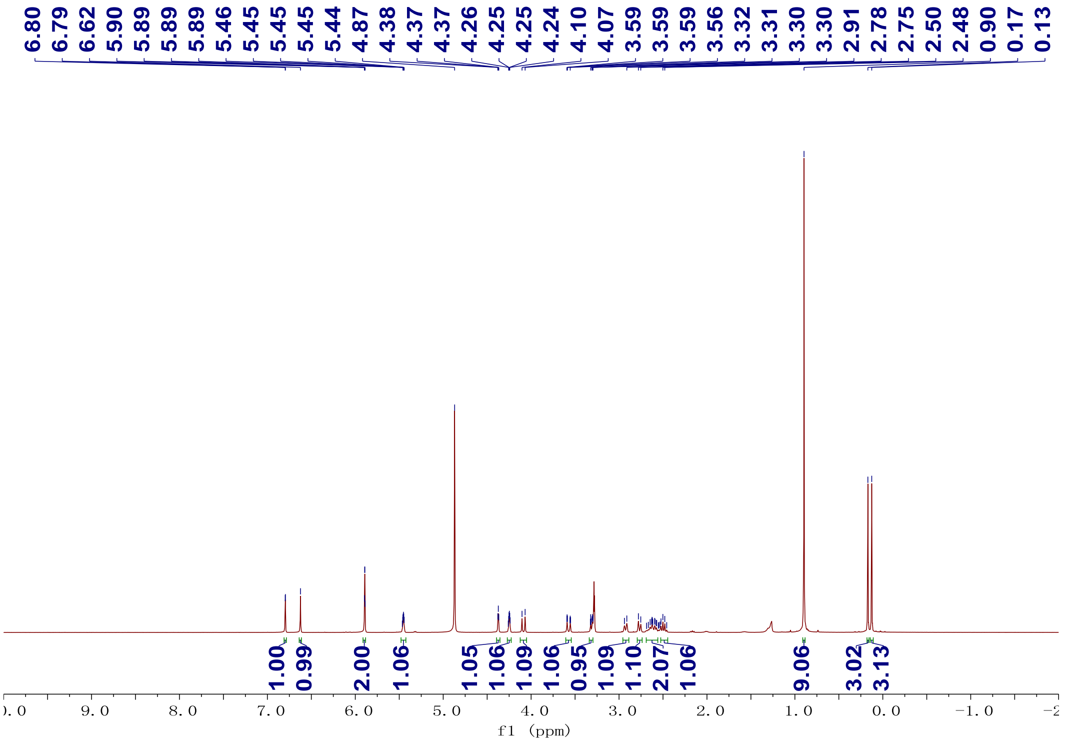

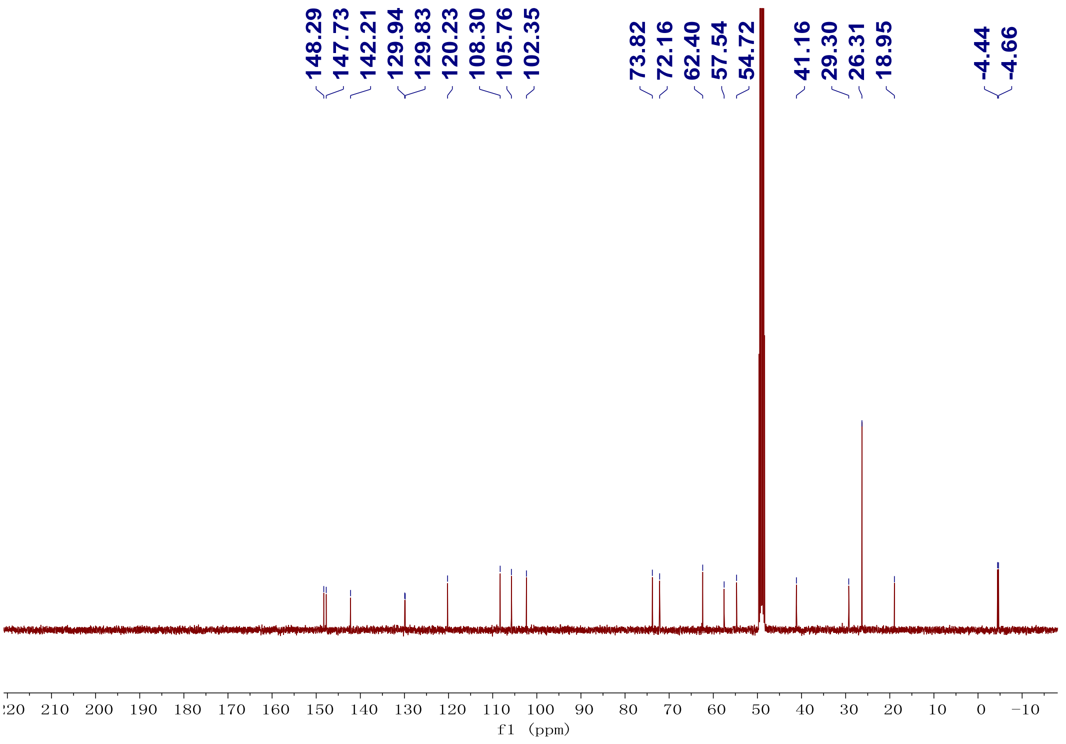

^{1}H NMR (400 MHz, 25 □, Methanol-*d*4), ^{13}C NMR (101 MHz, 25 □, Methanol-d_4) for **WFQ3-2**

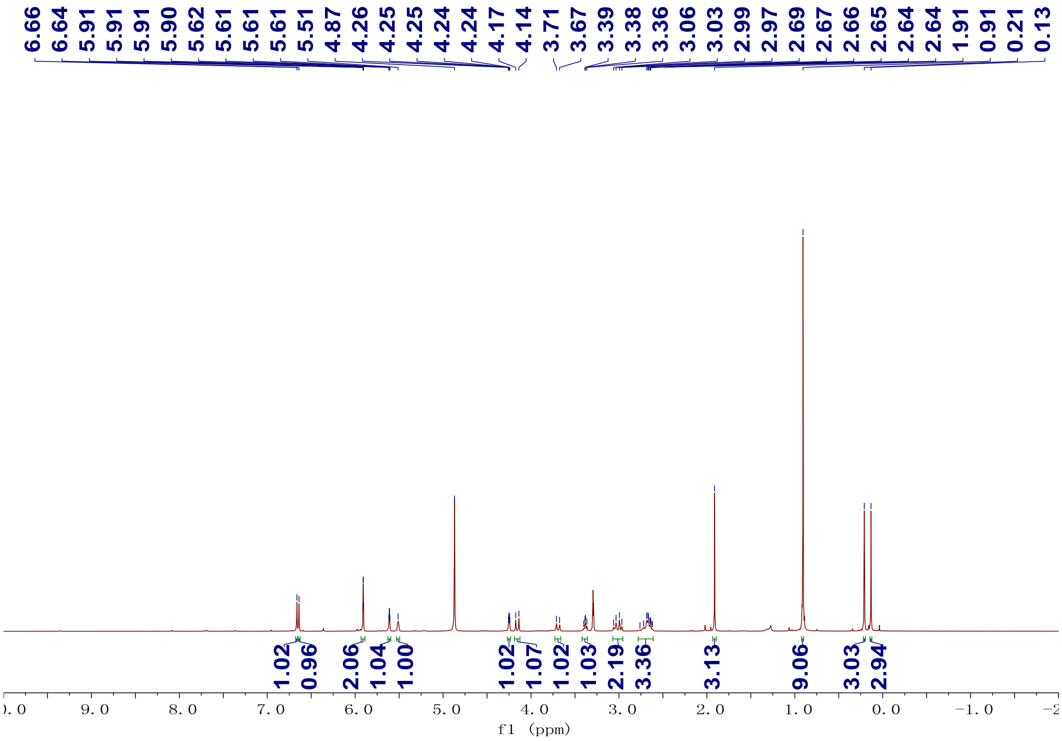

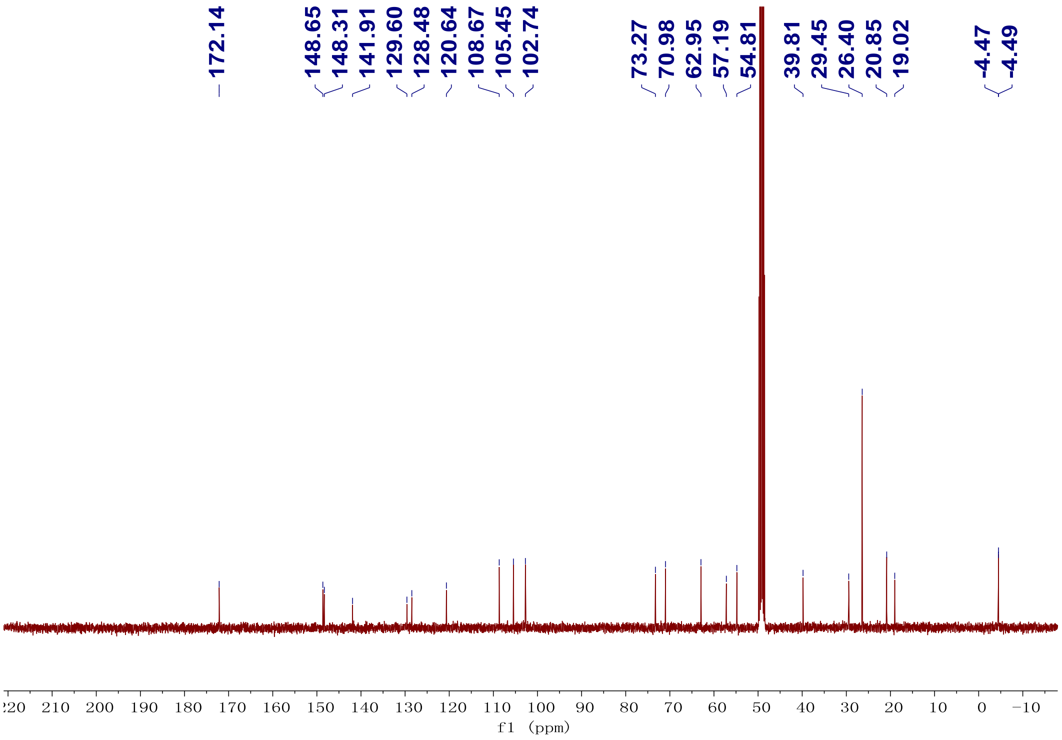

^{1}H NMR (400 MHz, 25 □, Methanol-*d*4), ^{13}C NMR (101 MHz, 25 □, Methanol-d_4) for **WFQ3-3**

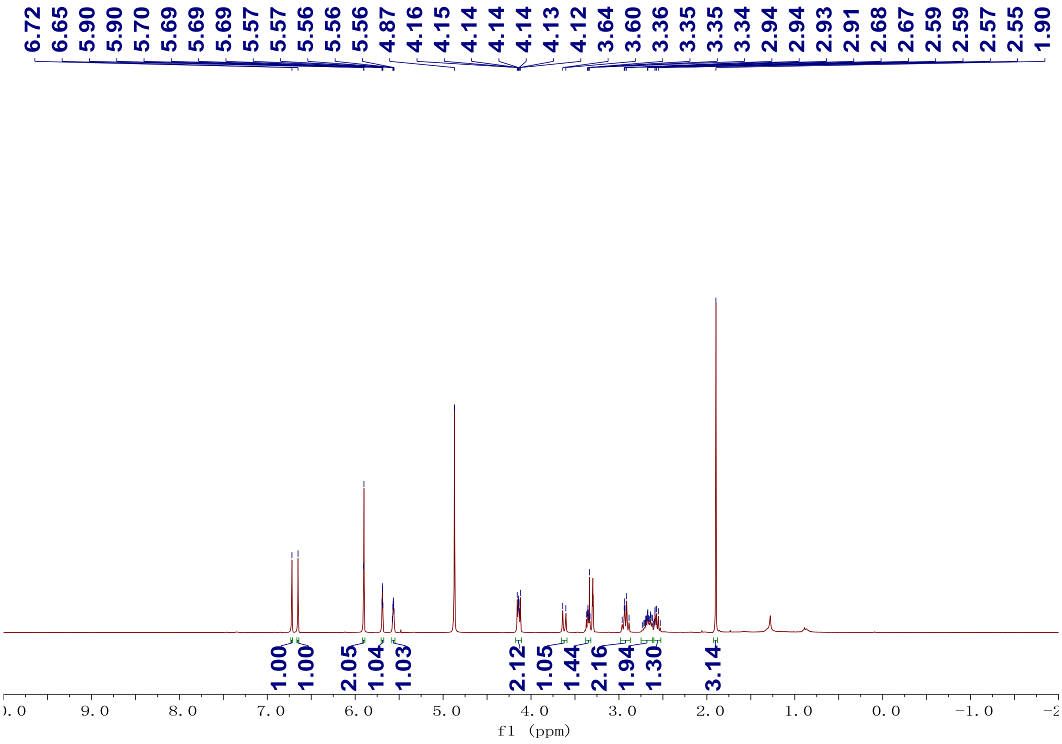

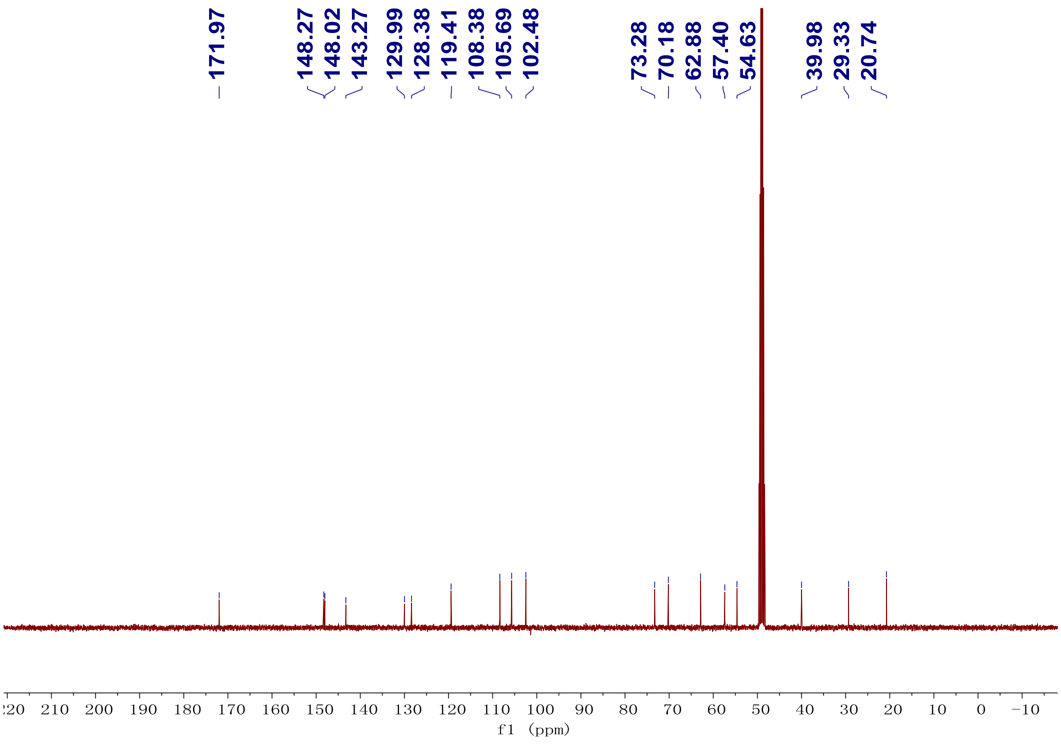

^{1}H NMR (400 MHz, 25 □, Methanol-*d*4), ^{13}C NMR (101 MHz, 25 □, Methanol-d_4) for **WFQ3-4**

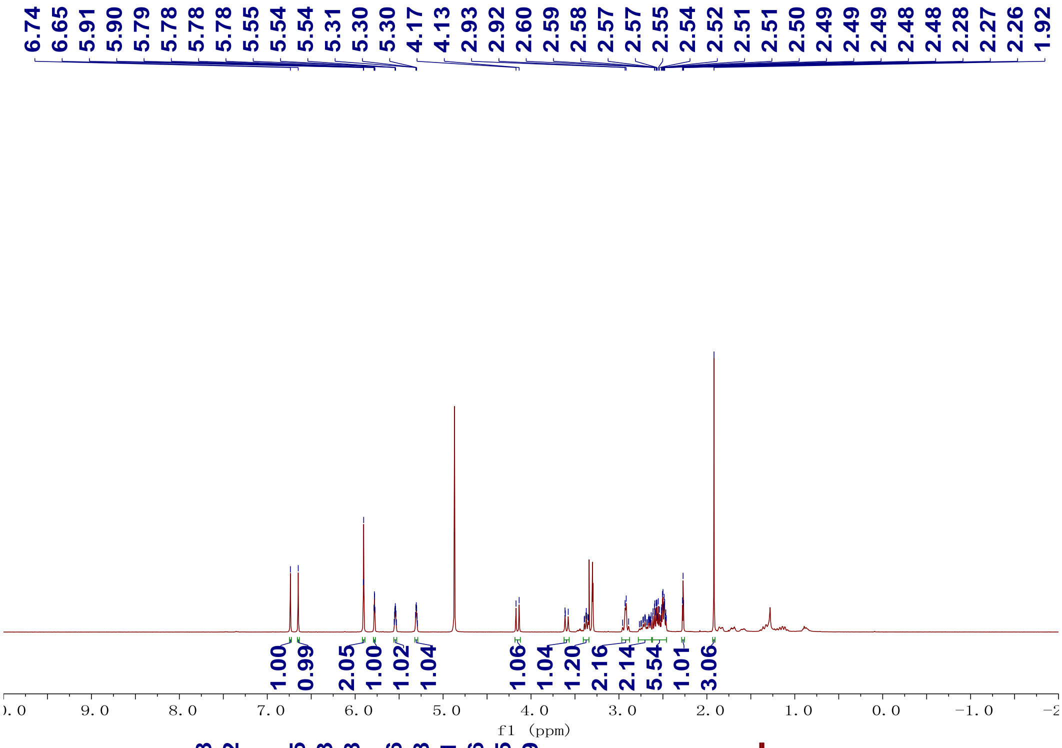

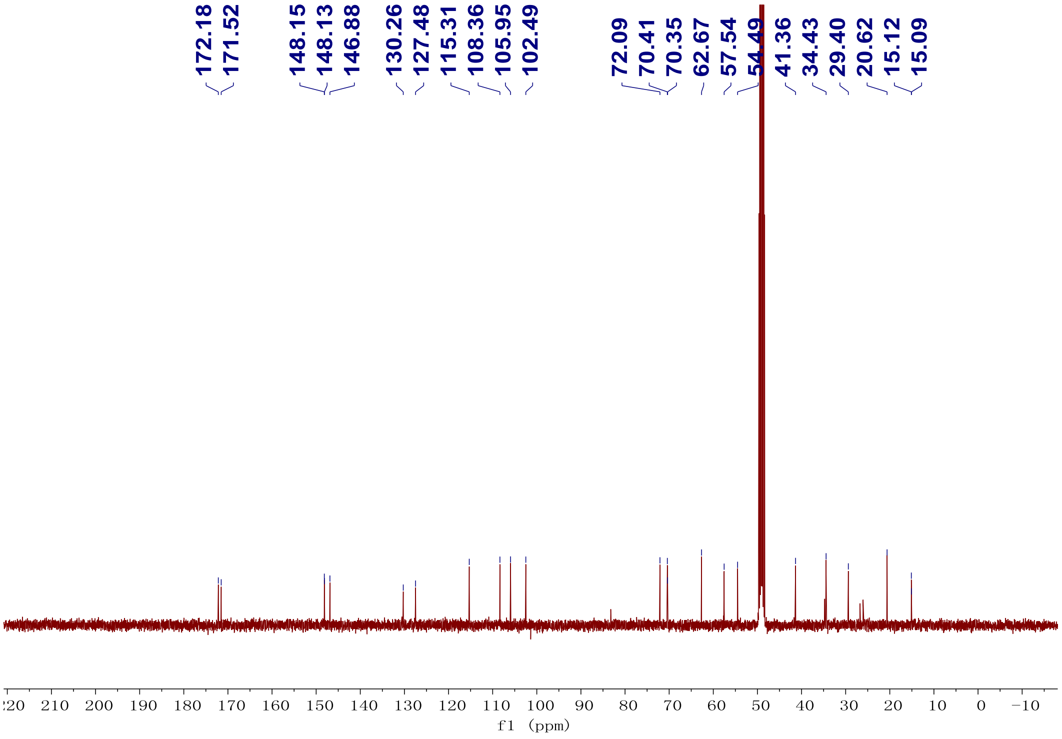

^{1}H NMR (400 MHz, 25 □, Methanol-*d*4), ^{13}C NMR (101 MHz, 25 □, Methanol-*d*$_4$) for **WFQ3-5**

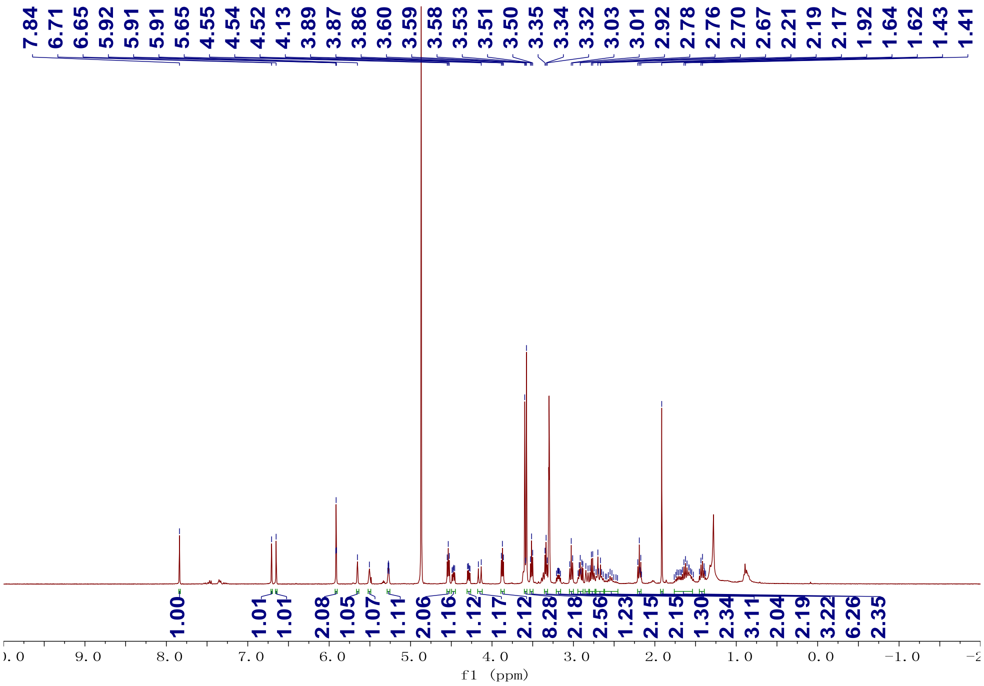

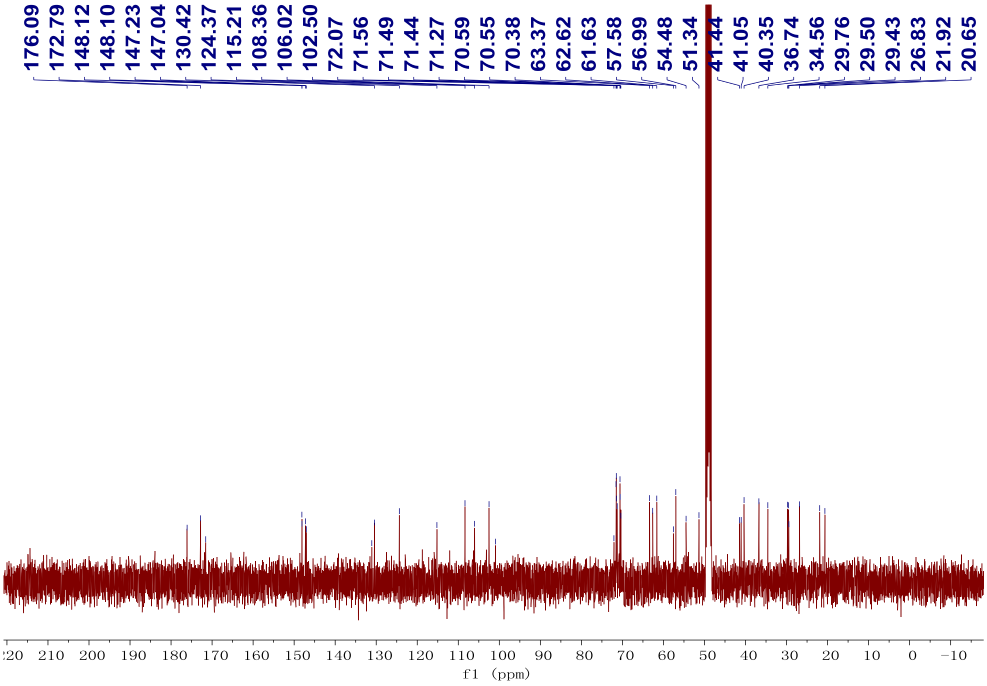

Supplemental tables

Table S1. Significant enrichment of terms from upregulated genes in graft-infiltrating $CD4^+$ T cells by GO analysis

NO	ID	Description	GO Class	pvalue	padj
1	GO:0006954	inflammatory response	BP	3.91E-28	6.37E-25
2	GO:0030593	neutrophil chemotaxis	BP	2.48E-16	2.02E-13
3	GO:0006955	immune response	BP	5.95E-15	3.23E-12
4	GO:0006935	chemotaxis	BP	1.01E-14	4.13E-12
5	GO:0002548	monocyte chemotaxis	BP	4.93E-12	1.61E-09
6	GO:0071222	cellular response to lipopolysaccharide	BP	2.11E-11	5.72E-09
7	GO:0008009	chemokine activity	MF	1.17E-10	2.73E-08
8	GO:0070098	chemokine-mediated signaling pathway	BP	1.49E-10	3.04E-08
9	GO:0070374	positive regulation of ERK1 and ERK2 cascade	BP	1.87E-10	3.39E-08
10	GO:0005125	cytokine activity	MF	3.67E-10	5.98E-08
11	GO:0048247	lymphocyte chemotaxis	BP	1.20E-09	1.78E-07
12	GO:0071347	cellular response to interleukin-1	BP	2.52E-09	3.43E-07
13	GO:0007204	positive regulation of cytosolic calcium ion concentration	BP	3.56E-09	4.46E-07
14	GO:0032729	positive regulation of interferon-gamma production	BP	4.66E-08	5.42E-06
15	GO:0004950	chemokine receptor activity	MF	1.54E-07	1.57E-05
16	GO:0090026	positive regulation of monocyte chemotaxis	BP	1.54E-07	1.57E-05
17	GO:0071346	cellular response to interferon-gamma	BP	2.75E-07	2.63E-05
18	GO:0016493	C-C chemokine receptor activity	MF	3.70E-07	3.14E-05
19	GO:0048020	CCR chemokine receptor binding	MF	3.70E-07	3.14E-05
20	GO:0019722	calcium-mediated signaling	BP	3.97E-07	3.14E-05
21	GO:0050729	positive regulation of inflammatory response	BP	4.45E-07	3.14E-05
22	GO:0032755	positive regulation of interleukin-6 production	BP	4.47E-07	3.14E-05
23	GO:0002437	inflammatory response to antigenic stimulus	BP	4.82E-07	3.14E-05
24	GO:0019957	C-C chemokine binding	MF	4.82E-07	3.14E-05
25	GO:0030595	leukocyte chemotaxis	BP	4.82E-07	3.14E-05
26	GO:0019956	chemokine binding	MF	7.89E-07	4.94E-05
27	GO:0060326	cell chemotaxis	BP	9.42E-07	5.68E-05
28	GO:0050135	NAD(P)+ nucleosidase activity	MF	2.06E-06	0.000115
29	GO:0061809	NAD+ nucleotidase, cyclic ADP-ribose generating	MF	2.06E-06	0.000115
30	GO:0031640	killing of cells of other organism	BP	3.33E-06	0.000181
31	GO:0019221	cytokine-mediated signaling pathway	BP	4.97E-06	0.000261
32	GO:0008285	negative regulation of cell population proliferation	BP	7.44E-06	0.000379
33	GO:0050728	negative regulation of inflammatory response	BP	9.69E-06	0.000478
34	GO:0032496	response to lipopolysaccharide	BP	1.04E-05	0.000499
35	GO:0032760	positive regulation of tumor necrosis factor production	BP	1.10E-05	0.000512
36	GO:0030335	positive regulation of cell migration	BP	1.36E-05	0.000613
37	GO:0010575	positive regulation of vascular endothelial growth factor production	BP	1.46E-05	0.000616
38	GO:0051092	positive regulation of NF-kappaB transcription factor activity	BP	1.48E-05	0.000616

39	GO:0017017	MAP kinase tyrosine/serine/threonine phosphatase activity	MF	1.48E-05	0.000616
40	GO:0043547	positive regulation of GTPase activity	BP	1.76E-05	0.000716
41	GO:0001525	angiogenesis	BP	1.90E-05	0.000754
42	GO:0030194	positive regulation of blood coagulation	BP	2.05E-05	0.000794
43	GO:0009617	response to bacterium	BP	2.42E-05	0.000916
44	GO:0045672	positive regulation of osteoclast differentiation	BP	2.55E-05	0.000944
45	GO:0048143	astrocyte activation	BP	2.76E-05	0.001
46	GO:0016525	negative regulation of angiogenesis	BP	2.89E-05	0.001024
47	GO:2000379	positive regulation of reactive oxygen species metabolic process	BP	3.58E-05	0.001213
48	GO:0045824	negative regulation of innate immune response	BP	3.65E-05	0.001213
49	GO:0051044	positive regulation of membrane protein ectodomain proteolysis	BP	3.65E-05	0.001213
50	GO:0032731	positive regulation of interleukin-1 beta production	BP	4.19E-05	0.00134
51	GO:0001774	microglial cell activation	BP	4.20E-05	0.00134
52	GO:0061844	antimicrobial humoral immune response mediated by antimicrobial peptide	BP	4.45E-05	0.001392
53	GO:0032689	negative regulation of interferon-gamma production	BP	5.68E-05	0.001719
54	GO:0071356	cellular response to tumor necrosis factor	BP	5.82E-05	0.001719
55	GO:0000188	obsolete inactivation of MAPK activity	BP	6.02E-05	0.001719
56	GO:0018149	peptide cross-linking	BP	6.02E-05	0.001719
57	GO:0048245	eosinophil chemotaxis	BP	6.02E-05	0.001719
58	GO:0034097	response to cytokine	BP	7.09E-05	0.001991
59	GO:0051019	mitogen-activated protein kinase binding	MF	7.55E-05	0.002083
60	GO:0001540	amyloid-beta binding	MF	7.83E-05	0.002125
61	GO:0030169	low-density lipoprotein particle binding	MF	9.35E-05	0.002495
62	GO:0002376	immune system process	BP	0.000107	0.002818
63	GO:0045766	positive regulation of angiogenesis	BP	0.00011	0.002845
64	GO:0097192	extrinsic apoptotic signaling pathway in absence of ligand	BP	0.000112	0.002845
65	GO:0043277	apoptotic cell clearance	BP	0.000138	0.003467
66	GO:0043065	positive regulation of apoptotic process	BP	0.000142	0.003503
67	GO:0006898	receptor-mediated endocytosis	BP	0.000162	0.003935
68	GO:0048146	positive regulation of fibroblast proliferation	BP	0.000191	0.004575
69	GO:0005041	low-density lipoprotein particle receptor activity	MF	0.000208	0.004774
70	GO:0046902	regulation of mitochondrial membrane permeability	BP	0.000208	0.004774
71	GO:0071223	cellular response to lipoteichoic acid	BP	0.000208	0.004774
72	GO:0032703	negative regulation of interleukin-2 production	BP	0.000233	0.005258
73	GO:0030217	T cell differentiation	BP	0.000248	0.005536
74	GO:0008330	protein tyrosine/threonine phosphatase activity	MF	0.000284	0.006158
75	GO:0051400	BH domain binding	MF	0.000284	0.006158
76	GO:0008305	integrin complex	CC	0.000316	0.006504
77	GO:0035924	cellular response to vascular endothelial growth factor stimulus	BP	0.000316	0.006504
78	GO:0048844	artery morphogenesis	BP	0.000316	0.006504
79	GO:0090023	positive regulation of neutrophil chemotaxis	BP	0.000316	0.006504
80	GO:0043524	negative regulation of neuron apoptotic process	BP	0.00032	0.006504

81	GO:0002237	response to molecule of bacterial origin	BP	0.000375	0.007351
82	GO:0004115	3',5'-cyclic-AMP phosphodiesterase activity	MF	0.000375	0.007351
83	GO:0070997	neuron death	BP	0.000375	0.007351
84	GO:0048286	lung alveolus development	BP	0.000405	0.007842
85	GO:0045089	positive regulation of innate immune response	BP	0.00042	0.007994
86	GO:0042127	regulation of cell population proliferation	BP	0.000422	0.007994
87	GO:0032757	positive regulation of interleukin-8 production	BP	0.000443	0.008293
88	GO:0048661	positive regulation of smooth muscle cell proliferation	BP	0.000464	0.008583
89	GO:0002430	complement receptor mediated signaling pathway	BP	0.000483	0.008733
90	GO:0045236	CXCR chemokine receptor binding	MF	0.000483	0.008733
91	GO:0050727	regulation of inflammatory response	BP	0.000496	0.008873
92	GO:0001968	fibronectin binding	MF	0.000546	0.009657
93	GO:0008360	regulation of cell shape	BP	0.000559	0.009777
94	GO:0008630	intrinsic apoptotic signaling pathway in response to DNA damage	BP	0.000576	0.00997
95	GO:0062023	collagen-containing extracellular matrix	CC	0.000589	0.010092
96	GO:0038024	cargo receptor activity	MF	0.000609	0.010158
97	GO:0097242	amyloid-beta clearance	BP	0.000609	0.010158
98	GO:0031663	lipopolysaccharide-mediated signaling pathway	BP	0.000618	0.010158
99	GO:0032743	positive regulation of interleukin-2 production	BP	0.000618	0.010158
100	GO:0035690	cellular response to drug	BP	0.000626	0.010189
101	GO:0048246	macrophage chemotaxis	BP	0.000754	0.012157
102	GO:0035335	peptidyl-tyrosine dephosphorylation	BP	0.000781	0.012351
103	GO:0055074	calcium ion homeostasis	BP	0.000781	0.012351
104	GO:0005149	interleukin-1 receptor binding	MF	0.00092	0.013995
105	GO:0031527	filopodium membrane	CC	0.00092	0.013995
106	GO:0050918	positive chemotaxis	BP	0.00092	0.013995
107	GO:0050995	negative regulation of lipid catabolic process	BP	0.00092	0.013995
108	GO:0050873	brown fat cell differentiation	BP	0.000974	0.014675
109	GO:0002523	leukocyte migration involved in inflammatory response	BP	0.001107	0.016165
110	GO:0032611	interleukin-1 beta production	BP	0.001107	0.016165
111	GO:0032966	negative regulation of collagen biosynthetic process	BP	0.001107	0.016165
112	GO:0002020	protease binding	MF	0.001112	0.016165
113	GO:0002250	adaptive immune response	BP	0.001125	0.016202
114	GO:1901224	positive regulation of NIK/NF-kappaB signaling	BP	0.001153	0.01647
115	GO:0006096	glycolytic process	BP	0.001197	0.016795
116	GO:0008138	protein tyrosine/serine/threonine phosphatase activity	MF	0.001197	0.016795
117	GO:0070498	interleukin-1-mediated signaling pathway	BP	0.001316	0.018313
118	GO:0005178	integrin binding	MF	0.001387	0.019131
119	GO:0048873	homeostasis of number of cells within a tissue	BP	0.001453	0.019737
120	GO:0010629	negative regulation of gene expression	BP	0.001455	0.019737
121	GO:0042176	regulation of protein catabolic process	BP	0.001549	0.020837
122	GO:0031234	extrinsic component of cytoplasmic side of plasma membrane	CC	0.001617	0.021583
123	GO:0001938	positive regulation of endothelial cell proliferation	BP	0.001725	0.022825

124	GO:0007613	memory	BP	0.001746	0.022918
125	GO:0035970	peptidyl-threonine dephosphorylation	BP	0.001806	0.023328
126	GO:0043011	myeloid dendritic cell differentiation	BP	0.001806	0.023328
127	GO:0004721	phosphoprotein phosphatase activity	MF	0.001857	0.023803
128	GO:0001974	blood vessel remodeling	BP	0.001907	0.024251
129	GO:0043154	negative regulation of cysteine-type endopeptidase activity involved in apoptotic process	BP	0.001954	0.024664
130	GO:0043032	positive regulation of macrophage activation	BP	0.002087	0.025941
131	GO:0090050	positive regulation of cell migration involved in sprouting angiogenesis	BP	0.002087	0.025941
132	GO:0016311	dephosphorylation	BP	0.002118	0.026126
133	GO:0051897	positive regulation of protein kinase B signaling	BP	0.002326	0.02847
134	GO:0002687	positive regulation of leukocyte migration	BP	0.002395	0.028671
135	GO:0034113	heterotypic cell-cell adhesion	BP	0.002395	0.028671
136	GO:0034620	cellular response to unfolded protein	BP	0.002395	0.028671
137	GO:0005044	scavenger receptor activity	MF	0.00245	0.029109
138	GO:0045429	positive regulation of nitric oxide biosynthetic process	BP	0.002652	0.031285
139	GO:0032874	positive regulation of stress-activated MAPK cascade	BP	0.002729	0.03174
140	GO:0070555	response to interleukin-1	BP	0.002729	0.03174
141	GO:0045121	membrane raft	CC	0.002804	0.032372
142	GO:0007229	integrin-mediated signaling pathway	BP	0.002937	0.033667
143	GO:0004114	3',5'-cyclic-nucleotide phosphodiesterase activity	MF	0.003091	0.034707
144	GO:0032691	negative regulation of interleukin-1 beta production	BP	0.003091	0.034707
145	GO:0050870	positive regulation of T cell activation	BP	0.003091	0.034707
146	GO:0006952	defense response	BP	0.003454	0.038291
147	GO:0010759	positive regulation of macrophage chemotaxis	BP	0.003481	0.038291
148	GO:0032753	positive regulation of interleukin-4 production	BP	0.003481	0.038291
149	GO:0010595	positive regulation of endothelial cell migration	BP	0.003837	0.041856
150	GO:0034612	response to tumor necrosis factor	BP	0.0039	0.041856
151	GO:0070527	platelet aggregation	BP	0.0039	0.041856
152	GO:0001666	response to hypoxia	BP	0.003908	0.041856
153	GO:0043027	cysteine-type endopeptidase inhibitor activity involved in apoptotic process	MF	0.004347	0.04537
154	GO:0043236	laminin binding	MF	0.004347	0.04537
155	GO:0043425	bHLH transcription factor binding	MF	0.004347	0.04537
156	GO:0045780	positive regulation of bone resorption	BP	0.004347	0.04537
157	GO:0008081	phosphoric diester hydrolase activity	MF	0.004397	0.045593
158	GO:0009968	negative regulation of signal transduction	BP	0.004697	0.048393
159	GO:0071480	cellular response to gamma radiation	BP	0.004825	0.049098
160	GO:1900745	positive regulation of p38MAPK cascade	BP	0.004825	0.049098

Table S2. Significant enrichment of terms from upregulated genes in graft-infiltrating CD8$^+$ T cells by GO analysis

NO	ID	Description	GO Class	pvalue	padj
1	GO:0006954	inflammatory response	BP	2.53E-13	2.93E-10
2	GO:0030593	neutrophil chemotaxis	BP	2.92E-09	1.69E-06
3	GO:0006955	immune response	BP	3.79E-08	1.46E-05
4	GO:0008285	negative regulation of cell population proliferation	BP	2.98E-07	7.30E-05
5	GO:0071222	cellular response to lipopolysaccharide	BP	3.15E-07	7.30E-05
6	GO:0070098	chemokine-mediated signaling pathway	BP	1.11E-06	0.000188
7	GO:0005125	cytokine activity	MF	1.14E-06	0.000188
8	GO:0050840	extracellular matrix binding	MF	3.42E-06	0.000495
9	GO:0070374	positive regulation of ERK1 and ERK2 cascade	BP	5.71E-06	0.000734
10	GO:0071346	cellular response to interferon-gamma	BP	8.02E-06	0.000929
11	GO:0032695	negative regulation of interleukin-12 production	BP	1.41E-05	0.001479
12	GO:2001237	negative regulation of extrinsic apoptotic signaling pathway	BP	1.80E-05	0.001737
13	GO:0008009	chemokine activity	MF	2.02E-05	0.001798
14	GO:0001525	angiogenesis	BP	2.70E-05	0.002236
15	GO:0048020	CCR chemokine receptor binding	MF	3.01E-05	0.002323
16	GO:0048844	artery morphogenesis	BP	4.88E-05	0.003534
17	GO:0048247	lymphocyte chemotaxis	BP	5.66E-05	0.003858
18	GO:0071347	cellular response to interleukin-1	BP	8.49E-05	0.004939
19	GO:2000379	positive regulation of reactive oxygen species metabolic process	BP	8.55E-05	0.004939
20	GO:0002237	response to molecule of bacterial origin	BP	8.96E-05	0.004939
21	GO:0050777	negative regulation of immune response	BP	8.96E-05	0.004939
22	GO:0002548	monocyte chemotaxis	BP	0.00011	0.005784
23	GO:0031640	killing of cells of other organism	BP	0.000124	0.005974
24	GO:0032689	negative regulation of interferon-gamma production	BP	0.000124	0.005974
25	GO:0071356	cellular response to tumor necrosis factor	BP	0.000176	0.008132
26	GO:0097192	extrinsic apoptotic signaling pathway in absence of ligand	BP	0.000213	0.009496
27	GO:0048661	positive regulation of smooth muscle cell proliferation	BP	0.000359	0.015382
28	GO:0043065	positive regulation of apoptotic process	BP	0.000394	0.0163
29	GO:0048010	vascular endothelial growth factor receptor signaling pathway	BP	0.000444	0.017725
30	GO:0043032	positive regulation of macrophage activation	BP	0.000515	0.019237
31	GO:0090050	positive regulation of cell migration involved in sprouting angiogenesis	BP	0.000515	0.019237
32	GO:1903672	positive regulation of sprouting angiogenesis	BP	0.000593	0.021459
33	GO:0006935	chemotaxis	BP	0.000706	0.02476
34	GO:0009968	negative regulation of signal transduction	BP	0.000805	0.027259
35	GO:0002437	inflammatory response to antigenic stimulus	BP	0.000871	0.027259
36	GO:0004181	metallocarboxypeptidase activity	MF	0.000871	0.027259
37	GO:0030595	leukocyte chemotaxis	BP	0.000871	0.027259
38	GO:0010575	positive regulation of vascular endothelial growth factor production	BP	0.000979	0.029837
39	GO:0043547	positive regulation of GTPase activity	BP	0.001011	0.030008
40	GO:0045766	positive regulation of angiogenesis	BP	0.001046	0.030281

41	GO:0035924	cellular response to vascular endothelial growth factor stimulus	BP	0.001095	0.030848
42	GO:0001227	DNA-binding transcription repressor activity, RNA polymerase II-specific	MF	0.00114	0.030848
43	GO:0002376	immune system process	BP	0.001145	0.030848
44	GO:0043542	endothelial cell migration	BP	0.00122	0.03211
45	GO:0045785	positive regulation of cell adhesion	BP	0.001341	0.034068
46	GO:0045672	positive regulation of osteoclast differentiation	BP	0.001353	0.034068
47	GO:0005925	focal adhesion	CC	0.001454	0.035339
48	GO:0001825	blastocyst formation	BP	0.001495	0.035339
49	GO:0016342	catenin complex	CC	0.001495	0.035339
50	GO:0030512	negative regulation of transforming growth factor beta receptor signaling pathway	BP	0.001594	0.035346
51	GO:0001968	fibronectin binding	MF	0.001646	0.035346
52	GO:0008157	protein phosphatase 1 binding	MF	0.001646	0.035346
53	GO:0022409	positive regulation of cell-cell adhesion	BP	0.001646	0.035346
54	GO:0032496	response to lipopolysaccharide	BP	0.001648	0.035346
55	GO:0005518	collagen binding	MF	0.00178	0.037483
56	GO:0002040	sprouting angiogenesis	BP	0.001976	0.039552
57	GO:0001938	positive regulation of endothelial cell proliferation	BP	0.001981	0.039552
58	GO:0034599	cellular response to oxidative stress	BP	0.001981	0.039552
59	GO:0043524	negative regulation of neuron apoptotic process	BP	0.002153	0.042255
60	GO:0043154	negative regulation of cysteine-type endopeptidase activity involved in apoptotic process	BP	0.002197	0.042401
61	GO:0001666	response to hypoxia	BP	0.002313	0.04269
62	GO:0004180	carboxypeptidase activity	MF	0.002345	0.04269
63	GO:0008626	granzyme-mediated apoptotic signaling pathway	BP	0.002471	0.04269
64	GO:0010828	positive regulation of glucose transmembrane transport	BP	0.002471	0.04269
65	GO:0043068	positive regulation of programmed cell death	BP	0.002471	0.04269
66	GO:0046581	intercellular canaliculus	CC	0.002471	0.04269
67	GO:0060982	coronary artery morphogenesis	BP	0.002471	0.04269
68	GO:0042327	positive regulation of phosphorylation	BP	0.002544	0.04269
69	GO:0051019	mitogen-activated protein kinase binding	MF	0.002544	0.04269
70	GO:0007623	circadian rhythm	BP	0.002676	0.044275
71	GO:0045177	apical part of cell	CC	0.002757	0.044969
72	GO:0030335	positive regulation of cell migration	BP	0.002908	0.045784
73	GO:0007179	transforming growth factor beta receptor signaling pathway	BP	0.002941	0.045784
74	GO:0006096	glycolytic process	BP	0.002972	0.045784
75	GO:0034115	negative regulation of heterotypic cell-cell adhesion	BP	0.003005	0.045784
76	GO:0051400	BH domain binding	MF	0.003005	0.045784
77	GO:0043433	negative regulation of DNA-binding transcription factor activity	BP	0.003523	0.048311
78	GO:0001913	T cell mediated cytotoxicity	BP	0.003588	0.048311
79	GO:0010884	positive regulation of lipid storage	BP	0.003588	0.048311
80	GO:0035455	response to interferon-alpha	BP	0.003588	0.048311
81	GO:0035456	response to interferon-beta	BP	0.003588	0.048311
82	GO:0038084	vascular endothelial growth factor signaling pathway	BP	0.003588	0.048311
83	GO:0042416	dopamine biosynthetic process	BP	0.003588	0.048311

84	GO:0070997	neuron death	BP	0.003588	0.048311
85	GO:0071498	cellular response to fluid shear stress	BP	0.003588	0.048311
86	GO:1902043	positive regulation of extrinsic apoptotic signaling pathway via death domain receptors	BP	0.003588	0.048311
87	GO:0043536	positive regulation of blood vessel endothelial cell migration	BP	0.003694	0.04917
